# β-Catenin Signaling Biases Multipotent Lingual Epithelial Progenitors to Differentiate and Acquire Specific Taste Cell Fates

**DOI:** 10.1371/journal.pgen.1005208

**Published:** 2015-05-28

**Authors:** Dany Gaillard, Mingang Xu, Fei Liu, Sarah E. Millar, Linda A. Barlow

**Affiliations:** 1 Department of Cell & Developmental Biology, and the Rocky Mountain Taste & Smell Center, University of Colorado Anschutz Medical Campus, Aurora, Colorado, United States of America; 2 Department of Dermatology, University of Pennsylvania School of Medicine, Philadelphia, Pennsylvania, United States of America; 3 Department of Cell & Developmental Biology, University of Pennsylvania School of Medicine, Philadelphia, Pennsylvania, United States of America; 4 Institute for Regenerative Medicine at Scott & White Hospital, Texas A&M University System Health Science Center, Temple, Texas, United States of America; Monell Chemical Senses Center, UNITED STATES

## Abstract

Continuous taste bud cell renewal is essential to maintain taste function in adults; however, the molecular mechanisms that regulate taste cell turnover are unknown. Using inducible Cre-lox technology, we show that activation of β-catenin signaling in multipotent lingual epithelial progenitors outside of taste buds diverts daughter cells from a general epithelial to a taste bud fate. Moreover, while taste buds comprise 3 morphological types, β-catenin activation drives overproduction of primarily glial-like Type I taste cells in both anterior fungiform (FF) and posterior circumvallate (CV) taste buds, with a small increase in Type II receptor cells for sweet, bitter and umami, but does not alter Type III sour detector cells. Beta-catenin activation in post-mitotic taste bud precursors likewise regulates cell differentiation; forced activation of β-catenin in these Shh^+^ cells promotes Type I cell fate in both FF and CV taste buds, but likely does so non-cell autonomously. Our data are consistent with a model where β-catenin signaling levels within lingual epithelial progenitors dictate cell fate prior to or during entry of new cells into taste buds; high signaling induces Type I cells, intermediate levels drive Type II cell differentiation, while low levels may drive differentiation of Type III cells.

## Author Summary

Taste is a fundamental sense that helps the body determine whether food can be ingested. Taste dysfunction can be a side effect of cancer therapies, can result from an alteration of the renewal capacities of the taste buds, and is often associated with psychological distress and malnutrition. Thus, understanding how taste cells renew throughout adult life, i.e. how newly born cells replace old cells as they die, is essential to find potential therapeutic targets to improve taste sensitivity in patients suffering taste dysfunction. Here we show that a specific molecular pathway, Wnt/β-catenin signaling, controls renewal of taste cells by regulating separate stages of taste cell turnover. We show that activating this pathway directs the newly born cells to become primarily a specific taste cell type whose role is to support the other taste cells and help them work efficiently.

## Introduction

The sense of taste is indispensable for feeding behavior. It informs the body whether food is harmful or nutritious, and thus is critical for regulating the intake of essential nutrients. Taste stimuli are detected in the oral cavity by taste buds, which are collections of neuroepithelial cells situated primarily in specialized taste papillae on the tongue surface. In rodents, fungiform papillae (FFP), each housing a single taste bud, are distributed on the anterior two thirds of the tongue, while a single circumvallate papilla (CVP), which contains several hundred taste buds, is situated at the posterior lingual midline. Regardless of location, each taste bud is a heterogeneous collection of ~60–100 elongate cells, which have both neural and epithelial characteristics: neural in that they transduce chemical signals, *i*.*e*., salt, sour, sweet, bitter, and umami (savory), into electrochemical signals which are transmitted via sensory afferents to the brain [1]; and epithelial, given their morphology and embryonic origin [2, 3](but see [4]) and the fact that taste cells are continuously renewed throughout life [5].

Taste cells are generated from cytokeratin (Krt) 5 and 14-expressing proliferative basal keratinocytes situated adjacent to taste buds [6]. The Krt14^+^ progenitor population also produces the non-taste or general epithelium, within which taste buds are embedded, and these cells undergo progressive differentiation, mirroring that of skin, to form the keratinized lingual epithelium [7]. Intriguingly, the pace of renewal of lingual epithelium is rapid, ~5–6 days [8, 9], comparable to that of intestinal epithelium (3–5 days) [10] and epidermis (8–10 days) [11, 12], while taste cells turn over significantly more slowly, on the order of 10–20 days [13–16]. Each taste bud comprises three morphological cell types; Type I cells likely serve a support function, may be salt detectors and are most prevalent within each bud [17]; Type III cells detect sour tastants and are least common; and Type II cells transduce sweet, bitter or umami tastes and occur at intermediate frequency [18–20]. Thus, in order to maintain the sense of taste, the Krt14^+^ progenitor population must: (1) produce both rapidly renewing, short-lived non-taste epithelium, and more slowly renewing, longer lived taste bud cells; and (2) generate the proper ratio of taste cell types I, II and III within each bud. Currently we have a limited understanding of how cell fate decisions are regulated within the lingual epithelial progenitor population.

Recently, an additional step in the taste bud cell lineage has been defined. Following their terminal cell division, Krt14^+^ daughters enter the basal compartment of taste buds as ovoid cells, turn on expression of Sonic hedgehog (Shh) [21], and subsequently differentiate into the different mature taste cell types [22]. Importantly, the frequencies with which Shh^+^ cells differentiate as Type I, II or III taste cells reflect the relative proportions of these cell types resident in the bud, *i*.*e*., I > II > III [22]. The point in the taste lineage at which postmitotic Shh^+^ precursor cell fate is regulated and the underlying molecular mechanisms are unknown.

One candidate is the Wnt/β-catenin pathway. We and others showed previously that Wnt/β-catenin signaling is both sufficient and required for embryonic taste bud development [23, 24]. Moreover, Wnt/β-catenin is a well-known regulator of renewing epithelia and epithelial appendages in adults, including skin, hair follicles and intestine, as well as neuroepithelium [25–30]. In the tongue, LacZ expression driven by the Wnt/β-catenin reporter allele, BATGAL, has revealed that β-catenin signaling is indeed active in cells both in and around adult taste buds, including basal keratinocytes, Shh^+^ precursor cells and in a subset of each of the 3 differentiated taste bud cell types [31], further strengthening the testable idea that this pathway regulates one or more steps in the taste lineage.

Here, using inducible, taste bud lineage-specific genetic manipulation, we define Wnt/β-catenin function in progressive steps of taste bud cell renewal. We show that activation of β-catenin within Krt14^+^/Krt5^+^ progenitors transiently accelerates proliferation, then forces these cells to exit the cell cycle and rapidly differentiate. However, rather than producing the entire spectrum of Krt14^+^ cell-derived fates, the induced daughter cells are fully diverted from differentiating as general lingual epithelium, and instead become taste cells. Significantly, these daughters differentiate almost exclusively into Type I taste cells—those purported to function primarily as support cells, and to a much smaller extent Type II receptor cells. The third taste cell type, Type III sour detectors, are never induced. Finally, we show that β-catenin activation in postmitotic Shh^+^ precursor cells within taste buds likewise influences taste cell type differentiation, driving these cells to acquire predominantly a Type I cell fate, but this effect appears to be non-cell autonomous as Shh^+^ daughter cells with activated β-catenin differentiate into Type II and III cells with a frequency no different from controls. Rather, in FFP taste buds, activated β-catenin appears to function primarily taste bud autonomously to locally affect Type I cell differentiation; while in the posterior CVP, Type I cell fate is also broadly promoted including in taste buds where β-catenin has not been stabilized. We postulate that the different origins of FFP and CV taste buds from ectoderm and endoderm, respectively [32], as well as significant differences in the structure of FF and CV papillae, may underlie these differential effects.

## Results

As Krt14^+^ basal cells give rise to both lingual epithelium and taste bud cells [6], and Krt5 and Krt14 are co-expressed by lingual basal keratinocytes [6, 33], we first confirmed that our doxycycline-mediated Krt5-driven induction system results in Cre-mediated reporter expression in basal keratinocytes, lingual epithelium and taste bud daughter cells. Krt5rtTA;tetOCre;R26RLacZ mice did not express β-galactosidase in the absence of doxycycline (dox), while Xgal^+^ cells were readily evident in lingual tissue of mice fed dox-supplemented chow (S1A and S1B Fig). Following 4 days of dox feeding of adult mice, the majority of epithelial cells around taste buds were Xgal^+^ in the posterior CVP and anterior FFP, while only a few Xgal^+^ cells were detected within taste buds (S1A_2_ and S1B_2_ Fig, white arrowheads). The number of Xgal^+^ cells in taste buds steadily increased with progressively longer dox treatment; after 4 weeks on dox chow, FFP and CVP taste buds were strongly Xgal^+^ (S1A_3–4_ and S1B_3–4_ Fig). Overall, the inducible system resulted in more rapid labeling of CVP taste buds, compared with those of the FFP. To accommodate these differences in efficacy in subsequent experiments, we assessed the CVP after 4 days and the FFP after 7 days of induction, when roughly comparable numbers of Xgal^+^ cells were evident within buds (compare S1A_2_ Fig CVP with S1B_3_ Fig FFP). To further confirm the efficiency of the inducible system, Krt5rtTA;tetOCre;Ctnnb1^(Ex3)fl/+^ mice were fed dox chow for 4 or 7 days, and tongue tissue examined via β-catenin immunofluorescence. Consistent with R26RLacZ reporter induction, in both FFP and CVP, nuclear β-catenin was elevated in mutant epithelial cells compared to controls (S1C–S1D Fig, white arrowheads). Thus, we established a reliable method for altering β-catenin gene function in adult mouse tongue epithelium.

### Activation of β-catenin in basal keratinocytes induces taste bud-like structures at the expense of non-taste epithelium

**Fig 1:**
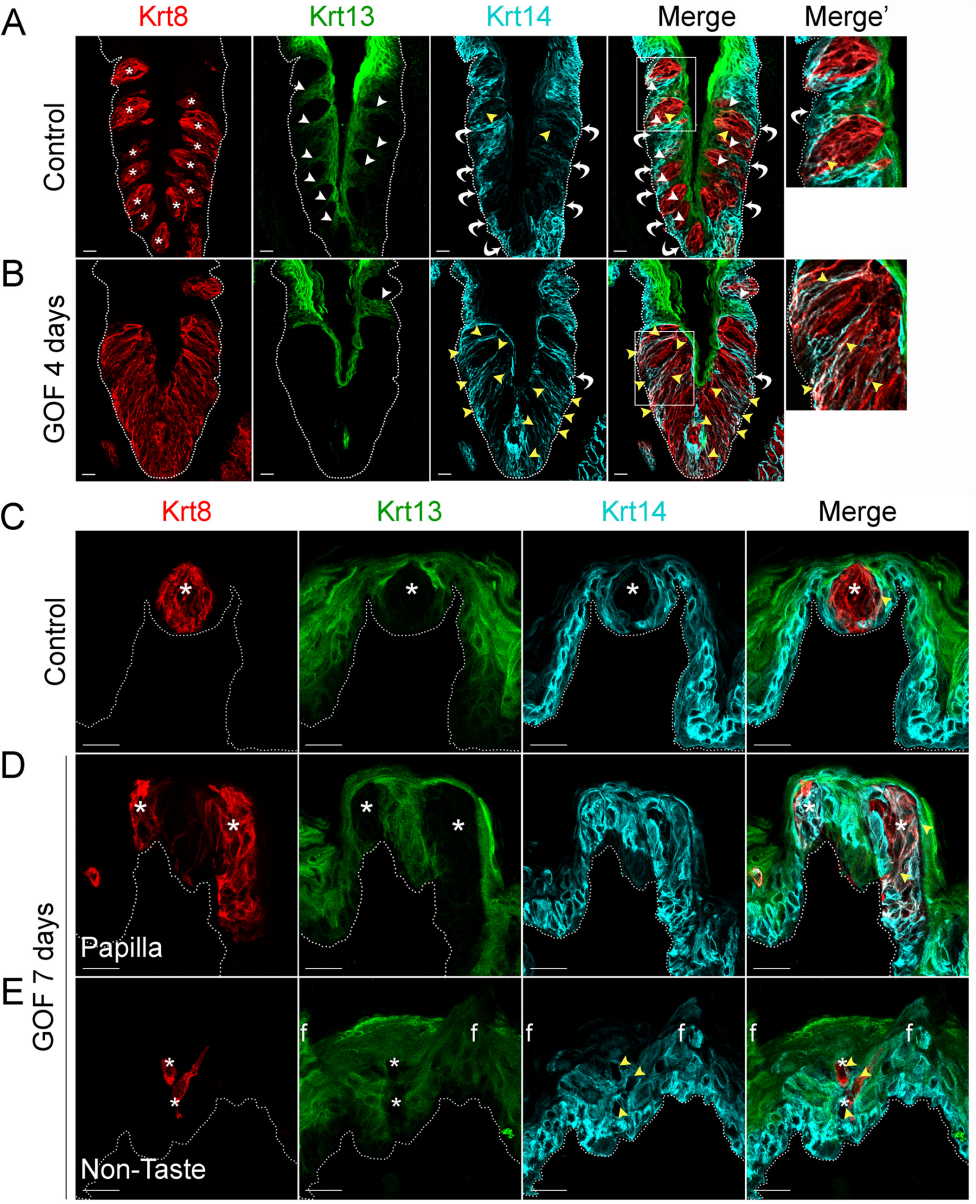
Stabilized β-catenin depletes progenitors (Krt14^+^) and causes lingual epithelial cells to differentiate as taste cells (Krt8^+^) at the expense of non-taste cells (Krt13^+^). Mutant (Krt5rtTA;tetOCre;Ctnnb1^**(Ex3)fl/+**^) and control mice were fed doxycycline-supplemented chow for 4 or 7 days, their tongues harvested, and CVP and anterior tongue sections immunostained for markers of taste cells (Krt8), non-taste squamous epithelium (Krt13), and basal progenitor cells (Krt14). **A**. In the CVP of control mice, distinct Krt8^**+**^ taste buds (red, * indicates a taste bud) are interspersed in the apical epithelium with Krt13^**+**^ postmitotic squamous keratinocytes (green, white arrowheads). Ovoid Krt14^**+**^ progenitor cells (cyan) are restricted to the basal epithelium (white bent arrows), although occasional elongate Krt8^**+**^ cells are also dimly Krt14^**+**^ (yellow arrowheads). **B**. In the CVP of Krt5rtTA;tetOCre;Ctnnb^**(Ex3)fl/+**^ mice, Krt8^**+**^ cells are greatly expanded, and Krt13^**+**^ cells typically found between taste buds are absent (compare white arrowheads in **A** and **B**). Ovoid Krt14^**+**^ basal cells are also slightly reduced (compare white arrows in **A** and **B**, and white arrows in **A** and **B** Merge’, while persistent Krt14 staining in the Krt8^**+**^ taste field is dramatically increased compared to controls (**A** and **B**, yellow arrowheads). **C**. In the anterior tongue of controls, each FFP houses a single Krt8^**+**^ taste bud (red, * indicates a taste bud) surrounded by Krt13^**+**^ squamous epithelium (green), while basal cells adjacent to taste buds and throughout the FFP epithelium are Krt14^**+**^ (cyan). **D**. In mutants with stabilized β-catenin, ectopic Krt8^**+**^ cell clusters occur in FFP (red), which are devoid of Krt13 (green; white asterisks), while many are Krt14^**+**^ (cyan, yellow arrowheads, white signal in merged panel). **E**. Krt8^**+**^ cells (red) are detected ectopically, interspersed among filiform papillae (“f”) of the non-taste lingual epithelium. These Krt8^**+**^ cells are Krt13-negative, and frequently Krt14^**+**^. Stack images are representative samples from 3 control and 3 mutant mice. Dotted line delineates the basement membrane. Scale bars = 20 μm.

In mice, Krt14^+^ basal keratinocytes of the CVP and FFP generate both non-taste lingual epithelium and taste bud cells [6], which express Krt13 [34] and Krt8 [33, 35], respectively. Thus, in the lingual epithelium these 3 keratins (Krt14, Krt13, and Krt8) serve as distinct markers for the 3 cell populations (progenitors, post-mitotic lingual epithelium, and taste buds, respectively), with minimal overlap (Fig 1). In contrast to the distinct, individual taste buds composed of elongate Krt8^+^ cells present in controls (Fig 1A, Krt8 red, asterisks), after 4 days on dox, the CVP epithelium of Krt5rtTA;tetOCre;Ctnnb1^(Ex3)fl/+^ mutants was occupied by a large, contiguous field of elongate Krt8^+^ cells (Fig 1B). When quantified via Krt8 immunofluorescence intensity (see Methods), we found a 2-fold increase in Krt8 signal in mutant CVP compared to controls (188.5 ± 28.2 vs 374.6 ± 21.3 in controls vs mutants; n = 3, p<0.0001, Student’s t-test). By contrast, Krt13^+^ squamous epithelial cells are found between taste buds in control CVP, but were virtually absent in mutant CVP (Fig 1A and 1B, Krt13 green, white arrowheads in control). In controls, Krt14 is expressed by proliferative keratinocytes situated at the basement membrane (Fig 1, Krt14 cyan, white bent arrows), whereas in mutants with stabilized β-catenin, Krt14^+^ basal cells were somewhat albeit not significantly diminished; Krt14 immunofluorescence intensity of cells at the basement membrane was decreased by 26% (110.4 ± 14.4 in controls vs 81.8 ± 8 in mutants; n = 3, p = 0.086, Student’s t-test). Instead, in mutants Krt14 immunostaining was evident abnormally in a subset of elongate Krt8^+^ cells in the expanded taste domain (Fig 1B, yellow arrowheads). Specifically, Krt14 immunofluorescence within the expanded taste epithelium was greatly increased (76.2 ± 15.4 in controls vs 181.9 ± 20.6 in mutants; n = 3, p = 0.0012, Student’s t-test). Overall, we observed a significant shift in the ratio between Krt14 immunofluorescence detected outside of (extragemmal) versus within (intragemmal) the expanded Krt8^+^ domain (median value = 1.6 in controls vs 0.4 in mutants; p<0.0001, Mann-Whitney test), suggesting the hypothesis that in response to activated β-catenin, progenitors are forced to rapidly differentiate into taste cells. Additionally, Krt14^+^ progenitors express the Sonic hedgehog (Shh) receptor and target gene, *Ptch1* (S2 Fig, control; [36, 37]), while in mutants, *Ptch1* expression is lost in the extragemmal compartment of the CVP (S2 Fig, GOF 4 days), further supporting the hypothesis that progenitor cells are reduced by activated β-catenin.

Similarly, in the anterior tongue, in contrast to the single Krt8^+^ taste bud resident in control FFPs (Fig 1C, asterisks), after 7 days of dox, multiple Krt8^+^ cell clusters were evident within existing FFPs (Fig 1D, asterisks). In mutants, we also detected numerous ectopic Krt8^+^ cell clusters among the spine-like filiform papillae of the non-taste epithelium (“f” in Fig 1E). Both types of ectopic clusters (in FFP or in non-taste epithelium) comprised elongate Krt8^+^ cells, which were also Krt13-immunonegative (Fig 1D and 1E, white asterisks), consistent with a taste fate. As in the CVP, Krt14^+^ basal keratinocytes were disorganized in both FFP and non-taste epithelium of the anterior tongue, and some ectopic Krt8^+^ cells were also abnormally Krt14^+^ (Fig 1D and 1E, yellow arrowheads).

**Fig 2:**
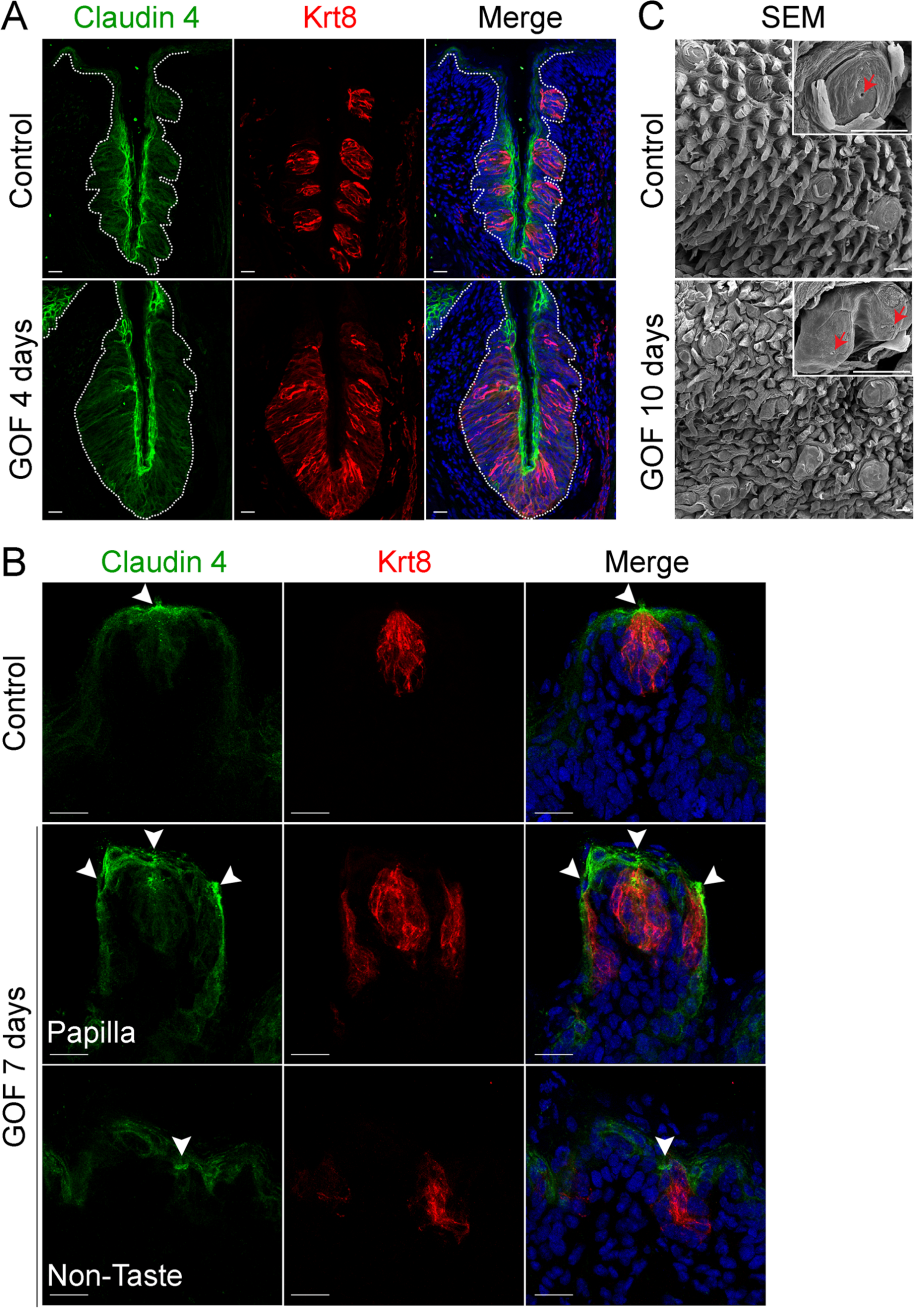
Expression of Claudin4 and evidence of taste pores suggests appropriate cell contacts are induced and maintained in mutant taste buds with stabilized β-catenin. Claudin4 is a tight junctional protein expressed specifically by control taste buds (A,B control). Claudin4 expression was maintained and expanded in mutant CVP taste epithelium (**A,** GOF 4 days, dotted line), and by ectopic taste buds in the FFP and non-taste epithelium (**B**, GOF 7 days, arrowheads). FFP in controls have a single taste pore shown via SEM (**C**, Control, red arrowhead), whereas in mutants treated with dox for 10 days, we encountered FFP with duplicated taste pores (**C**, GOF 10 days, red arrowhead). *NB*: red staining in the mesenchyme in **A** is due to non-specific secondary antibody binding. Nuclei were counterstained with DRAQ5 in blue. Three mice were used in each experimental group. Dotted line delineates the basement membrane. Scale bars = 20 μm in **A**,**B** and 30 μm in **C**.

To determine if taste cells induced by stabilized β-catenin maintained an organized epithelium, we assessed expression of Claudin4, a tight junction protein, which is associated with epithelial cell polarity and function [38, 39], and is expressed by taste bud cells [40, 41]. In control taste epithelium, Claudin4 is restricted primarily to taste cells, as well as to the squamous layer of the CVP trench and to the apical regions of FFP (Fig 2A and 2B)[40, 41]. Claudin4 expression was expanded, mirroring the expanded taste epithelium of the CVP in mice with stabilized β-catenin (Fig 2A, dotted line). In the anterior tongue, ectopic taste buds situated in the non-taste epithelium and within FFP were also appropriately Claudin4^+^, as Claudin4 expression was stronger in the apices of ectopic taste buds than in the rest of the epithelium (Fig 2B, arrowheads), indicating that these Krt8^+^ cells were properly polarized.

Taste cells within a bud terminate apically in specialized microvilli, which extrude into the taste pore, a small opening which allows the cells access to taste stimuli. This pore is considered the hallmark of a differentiated taste bud [42–45]. As expected in controls, a single taste pore was readily evident within FFP when tongues were examined via SEM (Fig 2C, Control, red arrowhead). However, in mutants treated with dox for 10 days, we found what appeared to be bifurcated taste papillae, each with a taste pore (Fig 2C, GOF 10 days, red arrowheads).

In sum, our data suggest the linked hypotheses that stabilized β-catenin causes Krt14^+^ epithelial progenitors to produce daughter cells committed to a taste fate (Krt8^+^) at the expense of a non-taste fate (Krt13^+^), potentially by forcing precocious differentiation of Krt14^+^ progenitors, as indicated by loss of *Ptch1* and a trend to diminished Krt14 by lingual progenitors, as well as by co-expression of Krt14 and Krt8 in elongate cells.

### Stabilized β-catenin alters progenitor proliferation, and accelerates specification of post-mitotic taste precursor cells

**Fig 3:**
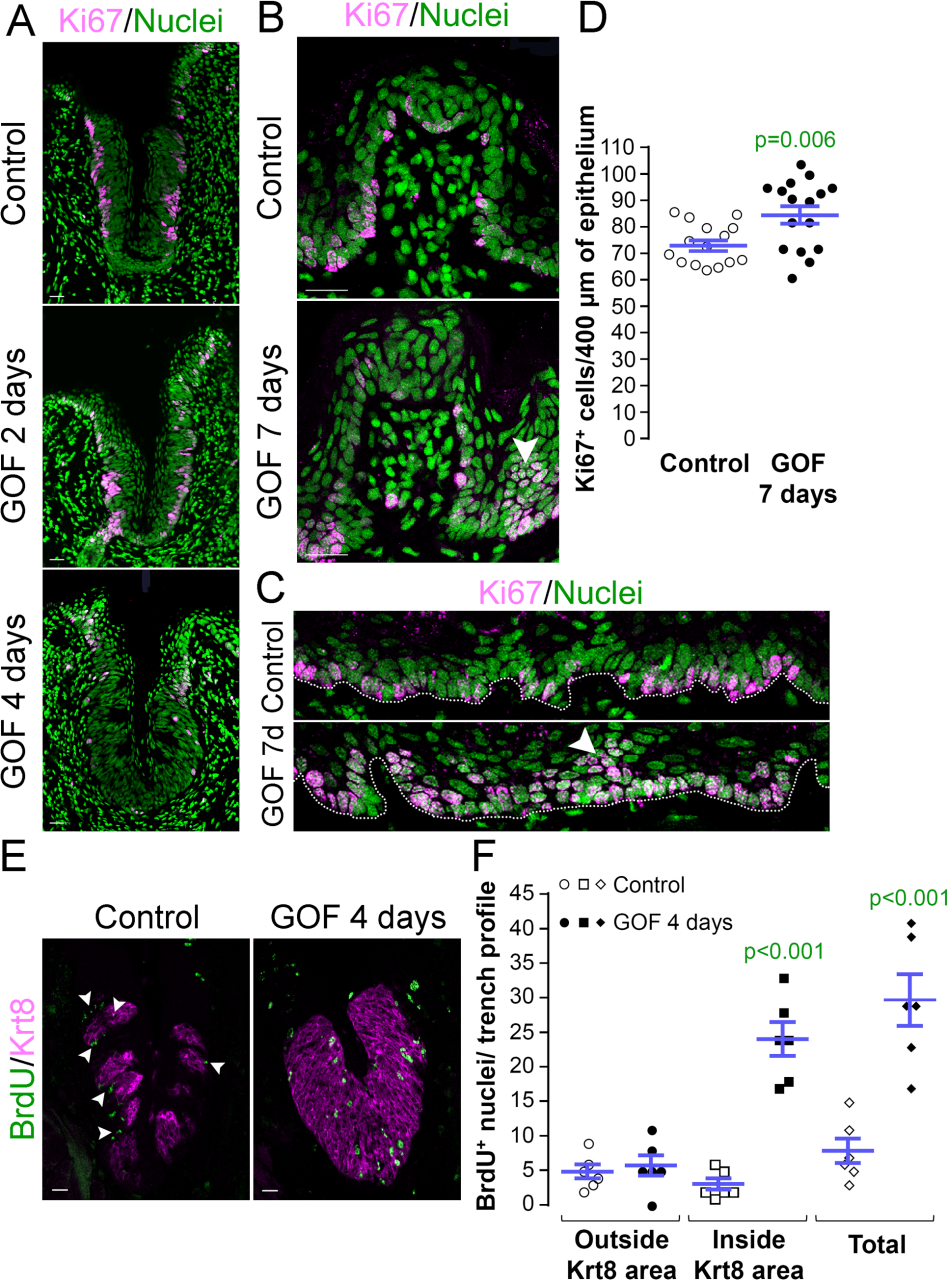
Stabilized β-catenin alters proliferation in anterior and posterior taste fields. A, GOF 2 days. After 2 days of dox, the proportion of Ki67^**+**^ basal keratinocytes in mutant CVP epithelium did not differ from controls (77.9 ± 2.3% vs. 76.2 ± 2.2% in controls vs. mutants; n = 3, p = 0.583, Student’s t-test). **A, GOF 4 days.** By day 4, proliferation in the mutant CVP was virtually abolished. **B**. In the FFP, the number of Ki67^**+**^ cells within the FFP epithelium are comparable in mutants and controls, but in mutants proliferation appears increased in epithelia at the FFP base (GOF 7 days vs control, white arrowheads). **C**. In controls, only cells in the basal layer of epithelium are Ki67^**+**^, while in mutants, multiple layers or clusters of cells were actively proliferating (GOF 7d, arrowhead). **D**. The number of Ki67^**+**^ basal cells per 400μm of epithelium is significantly increased in mutants. **E,F**. Mutant and control mice were injected with BrdU 48 hours before tongues were harvested after 4 days on dox chow. Postmitotic BrdU^**+**^ cells were detected in and around taste buds (Krt8^**+**^) in control CVP epithelium (white arrowheads), whereas β-catenin stabilization resulted in increased BrdU^**+**^ cells only within the expanded Krt8^**+**^ taste field. Nuclei were counterstained with Sytox Green. Stack images comprising 14 compressed 0.75 μm-thick optical sections are representative samples from 3 control and 3 mutant mice. Six CVP trenches and 15–16 sections of non-taste epithelium were analyzed per group. Data are represented as scatter plot (individual symbols), and mean ± SEM (blue bars). Student’s t-test. Dotted line delineates the basement membrane. Scale bars = 20 μm.

Taste bud cell turn-over occurs via proliferation of Krt14^+^ progenitors adjacent to taste buds, while taste cells within buds, as well as suprabasal Krt13^+^ epithelial cells, are post-mitotic. To determine if stabilized β-catenin altered proliferation of Krt14^+^ progenitor cells, we quantified the proliferative index (P.I.) of the basal epithelial compartment of the CVP (P.I. = Ki67^+^ basal cells/total basal cells; see methods of Nguyen et al., 2012 [46]). Following 2 days of dox, the P.I. of mutants and controls did not differ (Fig 3A, Ki67^+^ cells were 77.9 ± 2.3% vs. 76.2 ± 2.2% of all basal cells in controls vs. mutants, respectively; n = 3, p = 0.583, Student’s t-test), indicating that the same fraction of Krt14^+^ progenitors was actively cycling after 2 days of induction. After 4 days, however, proliferation was substantially reduced in the CVP of mutants compared with controls (Fig 3A), and this decrease was not attributable to increased cell death (0 ± 0 vs. 0.06 ± 0.06 TUNEL^+^ cells in controls vs. mutants after 4 days of dox; n = 3, p = 0.349, Mann-Whitney test).

Progenitor proliferation was also affected in the anterior lingual epithelium. However, in contrast to the diminished proliferative index observed for CVP epithelium, Ki67^+^ basal cells increased slightly, but significantly in the anterior tongue of mutants compared with controls. Specifically, clusters of Ki67^+^ cells were observed at the base of the FFP in the mutants (Fig 3B, white arrowhead), suggesting that β-catenin stabilization increased proliferation in specific compartments of the FFP. When examined more broadly, proliferation throughout the non-taste lingual epithelium increased significantly in the mutants, and we detected clusters of suprabasal proliferative cells that are not encountered in controls (Fig 3C and 3D, arrowhead).

To resolve these apparently opposing results, we reexamined progenitor proliferation in the CVP by monitoring the flow of newly born cells into taste epithelium. Mutant and control mice were fed dox for 2 days, then injected with BrdU 48 hours before tongues were harvested after a total of 4 days on dox chow. As expected, in control CVP epithelium, post-mitotic BrdU^+^ cells were detected in and around Krt8^+^ taste buds, consistent with the rapid rate of renewal of lingual epithelium [11] and slower turnover of taste bud cells [14–16] (Fig 3E, Control). By comparison, stabilized β-catenin caused a dramatic increase in the number of BrdU^+^ cells within the CVP epithelium (Fig 3E and 3F) due entirely to an increase in BrdU^+^ cells inside the expanded Krt8^+^ taste field (Fig 3E and 3F). Thus, the reduced proliferative index observed for the CVP after 4 days of dox (Fig 3A) is due to earlier accelerated and/or increased proliferation of the progenitor cells followed by *en masse* differentiation of the progenitors, consistent with the increased proliferation seen in response to activated β-catenin in the anterior tongue epithelium. In sum these data suggest that β-catenin signaling, in addition to causing precocious specification of new taste cells from Krt14^+^ progenitors, accelerates proliferation of these progenitors, and in the CVP, quickly depletes this population.

### Beta-catenin stabilization drives lingual epithelial cells to acquire predominantly a Type I taste cell fate

In mice, mature taste buds are made up of a heterogeneous collection of ~60 differentiated cells, including Type III cells which detect sour, Type II cells which detect sweet, bitter and umami tastes, and Type I cells which are thought to function as support cells and may be salt receptors. As stabilization of β-catenin in lingual progenitor cells results in expanded Krt8^+^ taste epithelium in CVP, FFP and anterior tongue non-taste epithelium, we next determined if all 3 taste cell types were similarly expanded using specific immunomarkers: SNAP25 for Type III [47], PLCβ2 for Type II [48], and NTPdase2 for Type I cells [49].

**Fig 4:**
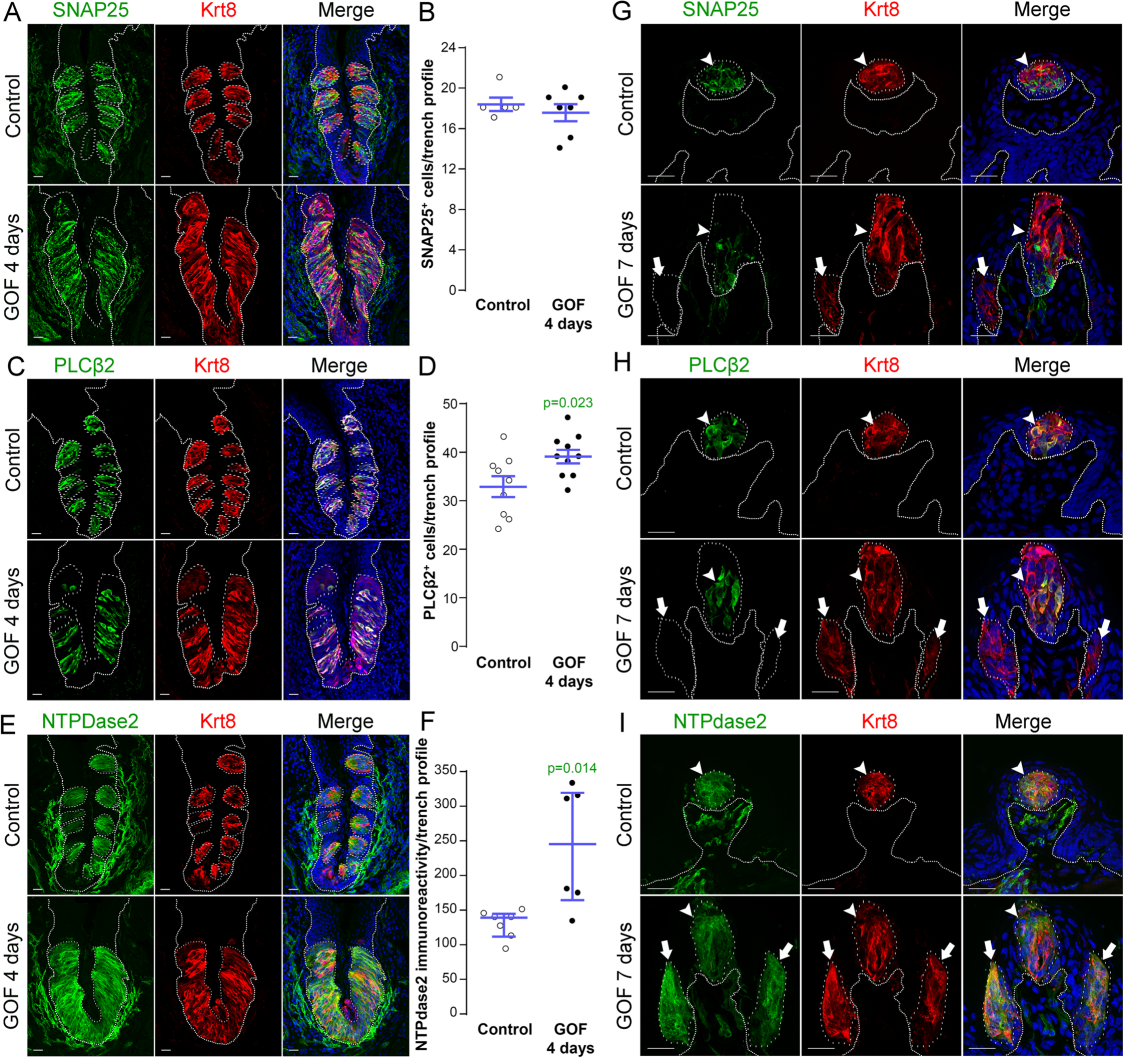
Stabilization of β-catenin in Krt5^+^ progenitors induces primarily Type I cell differentiation in CVP, FFP and anterior lingual epithelium. **A**,**B.** SNAP25^**+**^ Type III taste cells (green) were present in comparable numbers in the CVP of control and mutant mice fed dox chow for 4 days. Note that CVP innervation is also SNAP25^**+**^ (green) (Yang et al., 2000). **C**,**D**. PLCβ2^**+**^ Type II cells (green) were slightly increased in mutant CVP compared to controls. **E**,**F**. NTPdase2^**+**^ Type I cells (green) cannot be counted (see text), and thus were quantified via measurement of NTPdase2 corrected fluorescence intensity, revealing a 2-fold increase in NTPdase2^**+**^ signal in mutant compared to control CVP, accounting for the majority of expanded Krt8^**+**^ cells (red in all panels). **G**. In the anterior tongue of mice fed doxycycline chow for 7 days, endogenous Krt8^**+**^ taste buds within FFP (red, white arrowhead) contained SNAP25^**+**^ Type III cells (green), while ectopic Krt8^**+**^ taste buds (red, white arrow) were devoid of SNAP25^**+**^ cells. **H**. Endogenous FF taste buds house numerous PLCβ2^**+**^ Type II taste cells (green, white arrowhead), while ectopic PLCβ2^**+**^ cells were not detected in ectopic taste buds (* indicates a PLCβ2-negative ectopic taste bud). **I**. Ectopic taste buds within FFP of mutants (white arrows), like endogenous FF taste buds (white arrowhead) were always NTPdase2^**+**^ (green). Nuclei were counterstained with DRAQ5 in blue. Representative stack images are made of 14 compressed 0.75 μm-thick optical sections. N = 3 control and 3 mutant mice. **B**,**D**: Student’s t-test; **F**: Mann and Whitney test. Data are represented as a scatter plot (individual symbols), and mean ± SEM (blue bars), except **F** (median with interquartile range). Dotted line delineates the basement membrane. Scale bars = 20 μm.

In the CVP and FFP, Type I glial cells make up roughly half of the cells within each taste bud, while Type II and III cells each comprise ~10–30% of differentiated taste cells [19, 20]. Thus we reasoned that if β-catenin affected all taste cell fates similarly, then we would see proportionate increases in cell types I, II and III represented in the expanded Krt8^+^ domains. In the CVP, despite the robust increase in Krt8^+^ cells, however, the number of SNAP25^+^ Type III cells in mutant taste epithelium did not differ from controls (Fig 4A and 4B). Stabilization of β-catenin did result in a small increase in the number of PLCβ2^+^ Type II cells (Fig 4C and 4D), but this minimal gain was insufficient to account for the vast increase in Krt8^+^ taste cells (see Fig 1A). When we assessed Type I cells, however, we found that stabilized β-catenin induced a dramatic increase in NTPdase2^+^ epithelium in the CVP, which overlapped the expanded Krt8^+^ domain (Fig 4E). Because NTPDdase2 localizes to cell membranes, and Type I cells have elaborate sheet-like processes, it is not possible to accurately identify and therefore count individual NTPdase2^+^ Type I cells [22, 50, 51]. Instead, we used the density of NTPdase2 immunofluorescence as a proxy for the size of the Type I cell population (see Methods). We found a remarkable 2-fold increase in the intensity of NTPdase2 immunofluorescence (Fig 4F), as well as a significant expansion of the area of the CVP epithelium occupied by NTPdase2^+^ cells (S3A Fig). To verify that corrected fluorescence intensity is a reliable measure of the relative numbers of taste cells within buds, we applied this method to Type II cells, and found a significant correlation between the number and the fluorescence intensity of PLCβ2^+^ Type II cells (S3B Fig), and that the PLCβ2 fluorescence intensity levels in mutant CVP were slightly, albeit significantly, higher than in controls (S3B Fig), consistent with the small but significant increase in Type II cell number in mutants (see Fig 4D).

**Fig 5:**
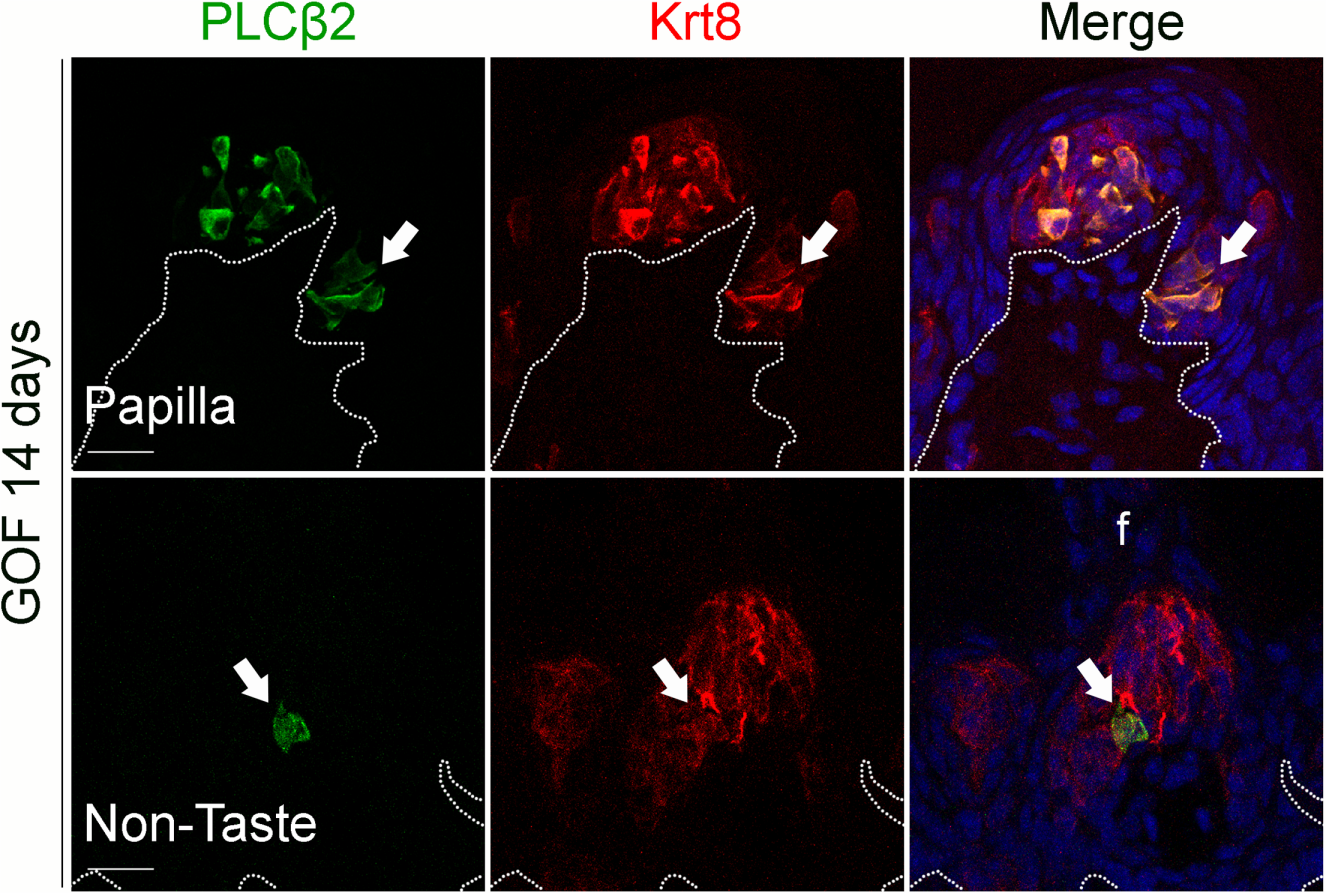
Prolonged stabilization of β-catenin in Krt5^+^ progenitors induces differentiation of a small number of Type II cells in ectopic taste bud-like structures in the anterior tongue. After induction with dox chow for 14 days, 5.8 ± 2% of anterior Krt8^**+**^ ectopic structures (total of 485 Krt8^**+**^ structures counted, 23 with PLCβ2, n = 3 mice) expressed PLCβ2 (green, white arrows). These ectopic structures never expressed the Type III cell marker SNAP25 (total of 356 Krt8^**+**^ structures counted, 0 with SNAP25^**+**^ cells, n = 3 mice). Nuclei were counterstained with DRAQ5 in blue. Representative stack images from 3 mutant mice. Dotted line delineates the basement membrane. Scale bars = 20 μm.

We next determined if Type I cells were also the dominant taste cell fate in ectopic Krt8^+^ clusters that formed in the anterior tongue in response to activated β-catenin. In the FFP of mice induced with dox for 7 days, SNAP25^+^ cells were not detected in ectopic Krt8^+^ clusters (Fig 4G, white arrow). In contrast to the CVP, PLCβ2^+^ Type II cells were not augmented in the anterior tongue, in that ectopic Krt8^+^ clusters in FFP were devoid of Type II cells (Fig 4H, white arrows). However, as we observed in the CVP using NTPdase2 to mark Type I cells, ectopic Krt8^+^ clusters within FFP were strongly NTPDase2^+^ (Figs 4I and S3C). Likewise, ectopic Krt8^+^ taste bud-like structures throughout the non-taste lingual epithelium were devoid of Type II and III taste cells after 7 days of dox; rather, ectopic Krt8^+^ taste buds were made up entirely of NTPdase2^+^ Type I cells (S4 Fig). Next, we analyzed the anterior tongues of mice induced for 14 days. Interestingly, and consistent with shorter term experimental results in the CVP epithelium, at this time point, 5.8 ± 2% of Krt8^+^ ectopic clusters housed elongate cells immunopositive for the Type II cell marker PLCβ2 (Fig 5, white arrows); however, in no instance did we observe cells expressing the Type III cell marker SNAP25 in ectopic Krt8 taste cell clusters (356 Krt8^+^ structures counted, 0 with SNAP25^+^ cells, n = 3 mice). In sum, stabilized β-catenin within lingual epithelial progenitors drives daughters to rapidly acquire a Type I, to a lesser extent a Type II, but not a Type III cell fate in both the CVP and anterior tongue, albeit over different time spans.

### Beta-catenin stabilization drives epithelial progenitors to generate Type I cells through a normal lineage progression

**Fig 6:**
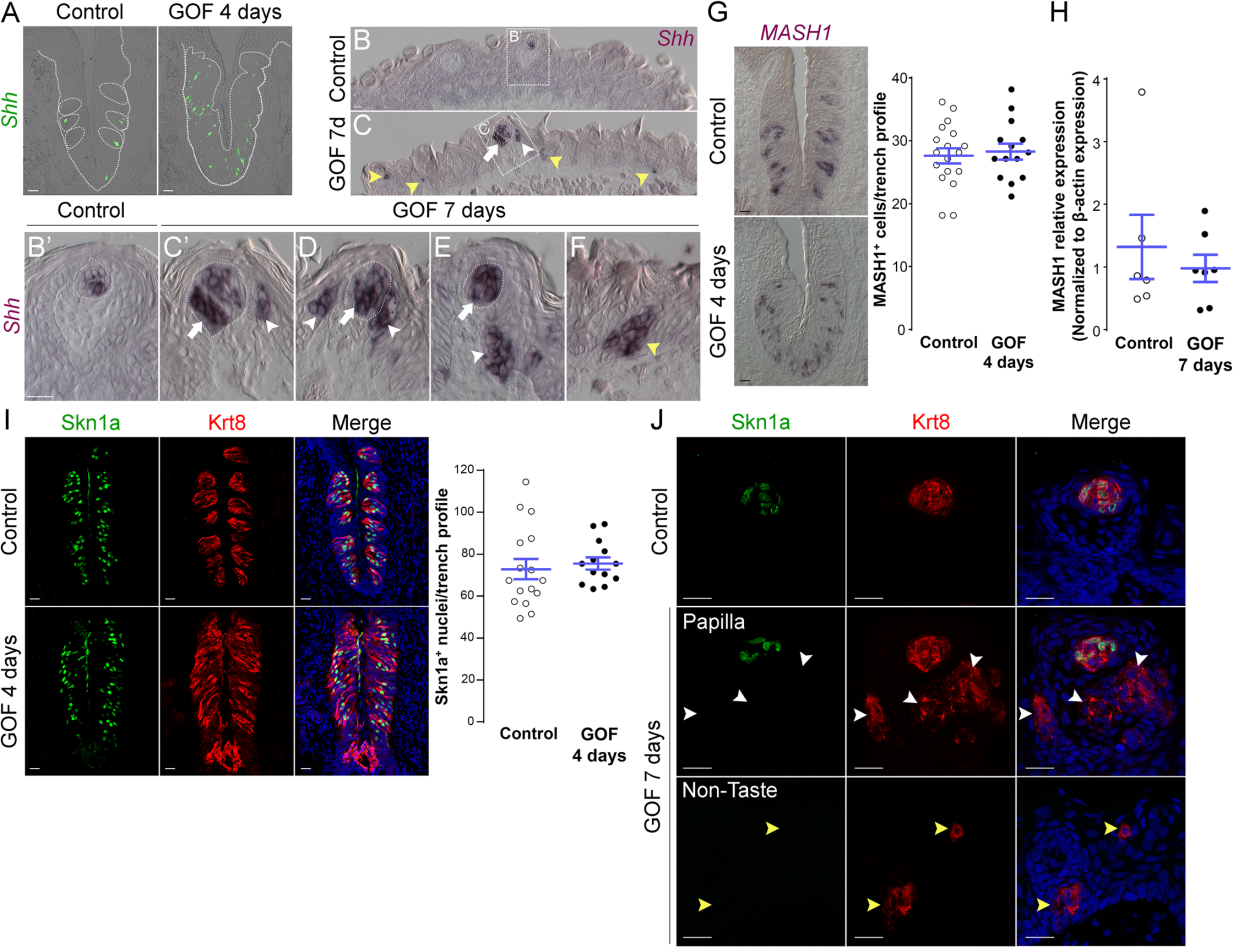
Stabilized β-catenin increases the number of Shh^+^ precursors of all three taste cell types, but does not impact specific precursors of Type II or III cells. **A**. In control mice, 1–3 Shh^**+**^ basal cells (green) are located in the basal compartment of each taste bud section (dashed circles), whereas in the CVP of mutant mice fed dox chow for 4 days, Shh^**+**^ cells (green) are increased and widespread in the expanded taste epithelium (dashed line). **B,B’**. In the anterior tongue of control mice, *Shh* ^**+**^ cells are restricted to FFP taste buds. **C-F**. In stabilized β-catenin mutants following 7 days on dox, in addition to endogenous *Shh* ^**+**^ cells (white arrows), ectopic *Shh* ^**+**^ cells are detected in FFP epithelium (white arrowheads in **C,C’,D,E**), as well as in non-taste epithelium adjacent to filiform papillae (yellow arrowheads in **C,F**). MASH1 and Skn-1a are expressed in subsets of basal cells within taste buds, and regulate the Type III and Type II cell lineage, respectively. CVP sections of control and mutant mice after 4 days on dox showed that *Mash1* ^***+***^ (**G**) and (**I**) Skn-1a^**+**^ (green) cell numbers were not altered by β-catenin stabilization. Real-time RT-PCR on the anterior tongue epithelium showed no difference in the expression of MASH1 between controls and mutants (**H**). **J**. After 7 days on dox chow, Skn-1a^**+**^ cells (green) were restricted to endogenous FFP taste buds in control and mutant mice (Krt8^**+**^ red, arrow). In mutants, Skn-1a^**+**^ cells were not detected in ectopic Krt8^**+**^ clusters in FFP epithelium (**J**, white arrowheads) or non-taste epithelium (**J**, yellow arrowheads). Nuclei were counterstained with DRAQ5 in blue. Dotted line indicates the basement membrane, and dashed circles indicate taste buds. Representative stack images and data from 3 control and 3 mutant mice. Data are represented as a scatter plot (individual symbols), and mean ± SEM (blue bars). Student’s t-test. Scale bars = 20 μm.

Shh is expressed specifically by post-mitotic basal cells within buds, and these basal cells are immediate precursors of all three taste cell types [21, 22]. Genetic stabilization of β-catenin in Krt5^+^ progenitors dramatically increased Shh^+^ precursor cells in the expanded CVP taste field compared to controls (Fig 6A). Similarly, in the anterior tongue of mutants (Fig 6B–6F), *Shh* expression was increased in endogenous FF taste buds (Fig 6C–6E, white arrows) and ectopically within the FFP epithelium (Fig 6C–6E, white arrowheads), as well as in ectopic locations throughout the non-gustatory epithelium of the anterior tongue (Fig 6C and 6F, yellow arrowheads). Our data suggest that forced β-catenin stabilization within epithelial progenitors promotes their specification to Shh^+^ taste bud precursors, which, although capable of giving rise to all 3 taste cell types in controls [22], ultimately biases these cells to differentiate into predominantly Type I taste cells.

A subset of basal cells within adult taste buds expresses *Mash1*, as well as *Shh*, and these basal cells have been proposed as precursors of Type III taste cells [52, 53]. We therefore determined if stabilized β-catenin affects specification of *Mash1* ^*+*^ precursors. In the CVP, *Mash1* ^*+*^ cell number in mutant epithelium did not differ from controls (Fig 6G). In the anterior tongue, while we were unable to detect Mash1 expression via anti-sense RNA or protein in mutants, real-time RT-PCR of peeled anterior tongue epithelium showed no significant difference in Mash1 expression in mutants compared to controls (Fig 6H), consistent with the fact that Type III taste cells are not induced by activated β-catenin (See Figs 4 and S4).

The POU domain transcription factor Skn-1a (POU2f3) is expressed by a sub-population of intragemmal basal cells and by Type II taste cells, and is required for Type II cell fate [54]. Therefore, we explored if stabilized β-catenin regulates the expression of Skn-1a. In contrast to the slight increase in Type II cells (see Figs 4D and 5), the number of Skn-1a^+^ cells was unchanged in mutant CVP (Fig 6I), suggesting that β-catenin stabilization may increase the number of Type II cells independently of Skn-1a. Consistent with the absence of Type II taste cells in ectopic Krt8^+^ clusters in the anterior tongue at 7 days, we did not encounter ectopic Skn-1a^+^ cells in the FFP or non-taste epithelium (Fig 6J).

### Beta-catenin stabilization in postmitotic Shh^+^ precursors biases taste cell fate

Broad activation of β-catenin in Krt5^+^ keratinocytes drives these progenitors to become Shh^+^ taste precursor cells, which in turn differentiate primarily into Type I, and infrequently, Type II taste cells (Figs 1 and 3–5). These data establish a role for β-catenin in selecting the fate of epithelial cells derived from Krt5^+^ progenitors, but left open if β-catenin stabilization within Shh^+^ taste precursors, once these cells have entered taste buds, affects cell fate selection. Thus, we induced stabilized β-catenin only in Shh^+^ basal cells in ShhCreERT2;Ctnnb1^(Ex3)fl/+^;R26R-YFP mice, and compared the relative proportions of Type I, II and III taste cells with those of controls (ShhCreERT2;R26R-YFP).

**Fig 7:**
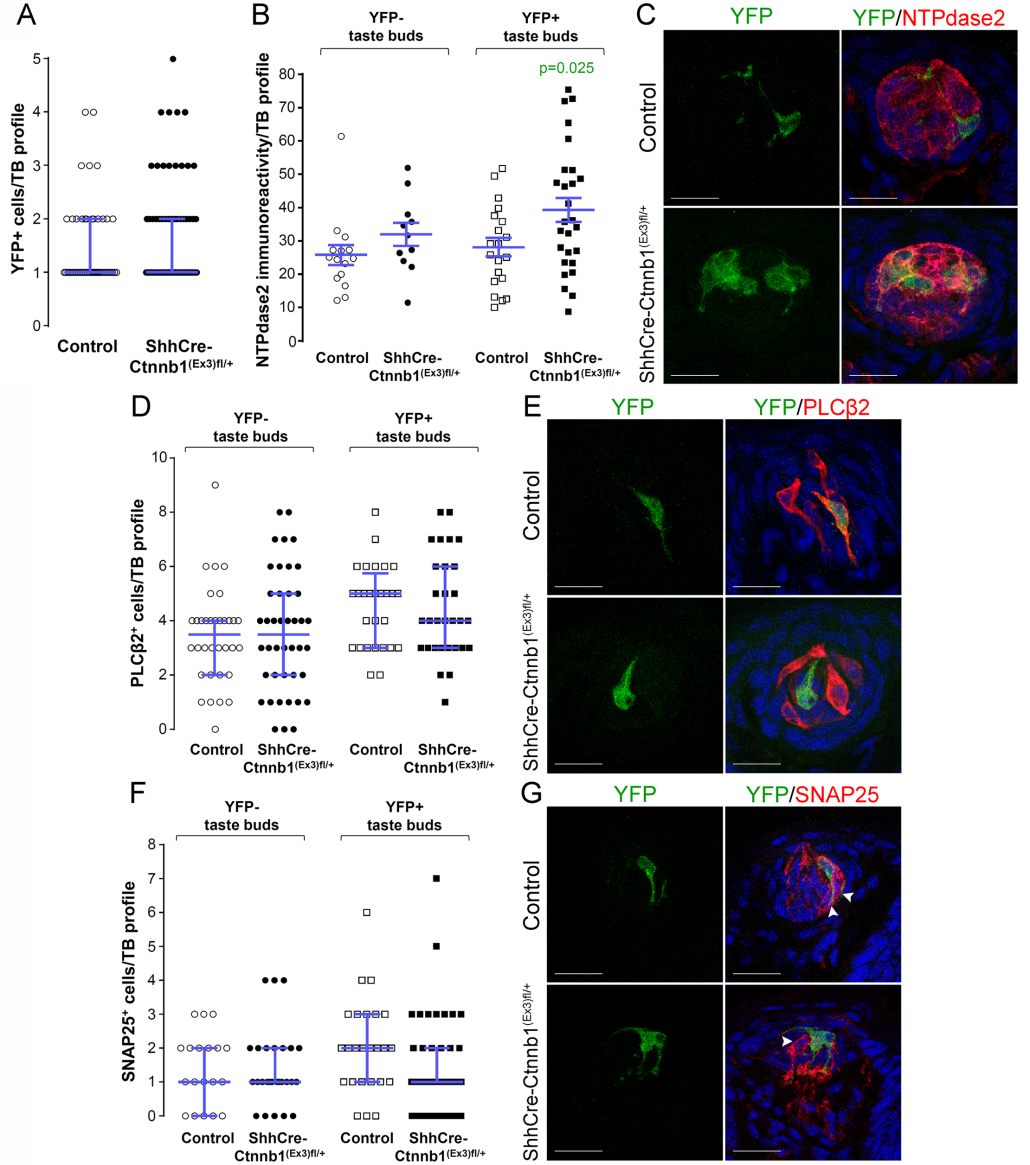
Stabilization of β-catenin in Shh^+^ precursor cells biases FFP taste cell fate. ShhCreERT2;Ctnnb^**(Ex3)fl/+**^;R26R-YFP mutant mice and their control counterparts (ShhCreERT2;R26R-YFP) were gavaged with tamoxifen daily for 8 days, and tongues harvested 14 days after the last dose. **A.** The number of Shh-descendant taste cells labeled with YFP did not differ between mutants and controls. **B,C**. In mutants, corrected NTPdase2^**+**^ immunofluorescence intensity of Type I cells was significantly increased in YFP^**+**^ taste buds, but not YFP^**-**^ taste buds. **D, E.** Overall the total number of Type II cells did not differ between mutants and controls, nor did the number of Type III cells (**F,G**). Representative stack images and data from 4–5 control and 4–6 mutant mice. **A**: 53 vs 72 YFP^**+**^ taste bud profiles from 6 control mice vs 6 mutant mice, respectively; **B**: 15 vs 11 YFP^**-**^ taste bud profiles and 20 vs 27 YFP^**+**^ taste bud profiles from 4 control mice vs 4 mutant mice, respectively; **D**: 34 vs 42 YFP^**-**^ taste bud profiles and 28 vs 30 YFP^**+**^ taste bud profiles from 4 control mice vs 4 mutant mice, respectively; **F**: 19 vs 27 YFP^**-**^ taste bud profiles and 25 vs 42 YFP^**+**^ taste bud profiles from 5 control mice vs 6 mutant mice, respectively. Mann & Whitney test, except **B** (Student’s t-test). Data are represented as scatter plots (individual symbols), and median with interquartile range (blue bars), except **B** (mean ± SEM). Nuclei are counterstained with DRAQ5 (blue). Scale bars = 20 μm.

In the FFP of the anterior tongue, the number of YFP^+^ cells per labeled taste bud did not differ between mutants and controls (Fig 7A); (although the overall percentage of fungiform taste buds housing YFP^+^ cells was higher in mutants, S5A Fig). We next asked if the relative proportions of the 3 taste cell types within lineage-labeled taste buds were altered by stabilized β-catenin within Shh^+^ precursors. We reasoned that if β-catenin activation within Shh^+^ cells intrinsically biased their differentiation to a Type I fate, then the proportions of Type II and/or Type III taste cells differentiating from Shh^+^ cells would be reduced, while Type I cells would be increased. As was the case for stabilized β-catenin in the Krt5^+^ population, NTPdase2-IR of Type I cells was significantly increased in YFP^+^ taste buds of mutant mice (Fig 7B and 7C). However, the number of Type II (Fig 7D and 7E), and Type III cells (Fig 7F and 7G) resident in YFP^+^ buds did not differ between mutants and controls. Importantly, Type I cells were significantly changed exclusively in YFP^+^ taste buds, and not in YFP^-^ taste buds (Fig 7B and 7C), indicating that the control of taste cell differentiation by β-catenin in the FFP is local, *i*.*e*., restricted to taste buds housing induced YFP^+^ mutant cells.

To determine if this shift in cell fate was due to cell autonomous effects of activated β- catenin within lineage labeled Shh^+^ cells, we compared the proportions of taste cell types among the YFP^+^ Shh-descendent cells in mutant versus controls. Due to the contorted morphology of Type I cells, however, we could not identify individual YFP^+^ Type I cells (see Miura et al., 2014 [22]), and thus could not ascertain if the overall increase in Type I cells (see Fig 7B and 7C) was due to cell-specific activation of β-catenin. However, Shh-descendent Type II and III cells were easily tallied, and we found that cell autonomous activation of β-catenin had no impact on Type II or III cell fate (S1 Table). Thus, our data suggest that activation of β-catenin within Shh^+^ cells inside taste buds acts by as-yet-to be identified local mechanisms to promote Type I cell differentiation.

**Fig 8:**
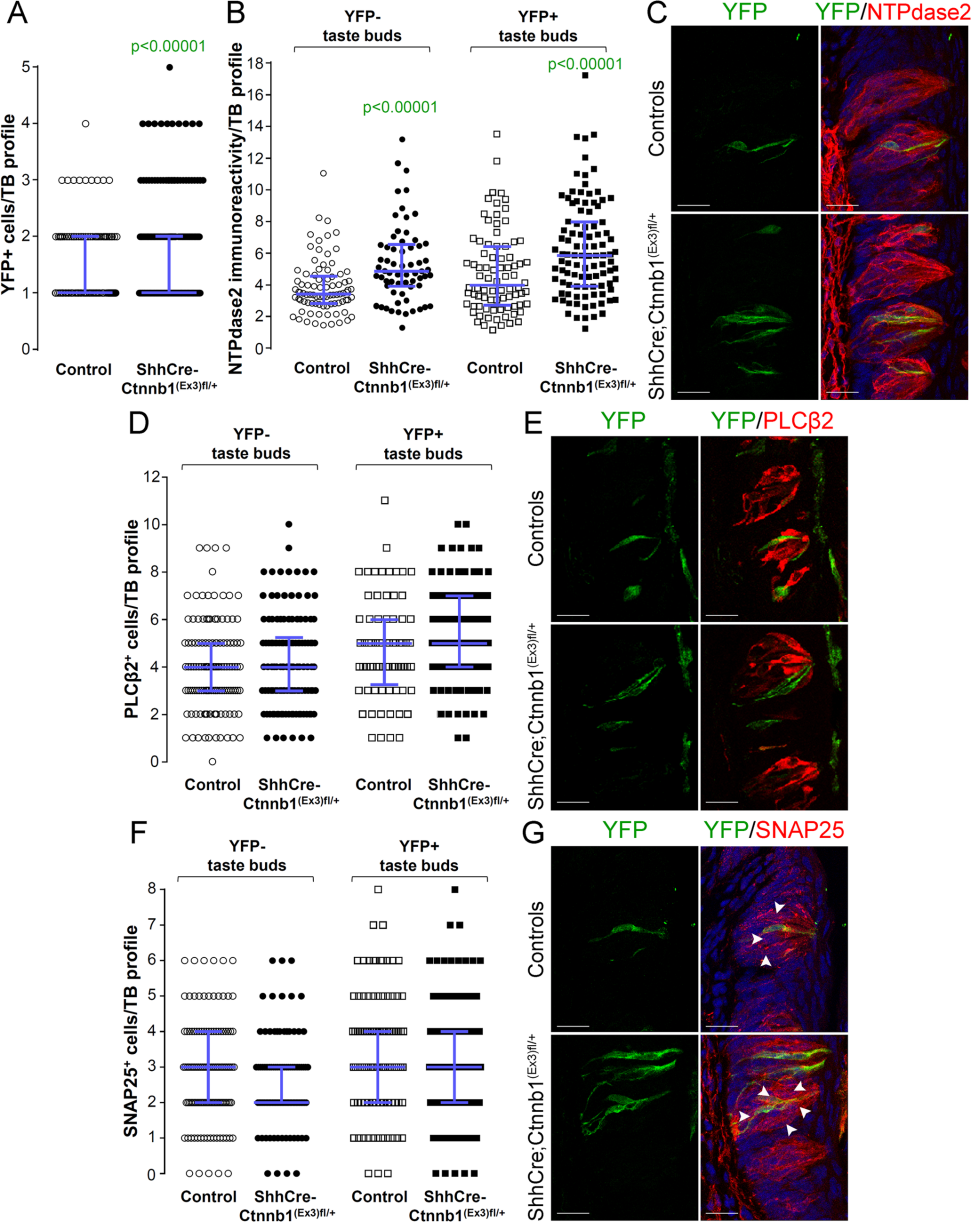
Beta-catenin stabilization in Shh^+^ cells biases taste cell fate in the CVP, both taste bud autonomously and indirectly. Beta-catenin stabilization in Shh^**+**^ precursors significantly increased the number of Shh- descendant taste cells labeled with YFP in mutant mice compared with controls (**A**). Overall the total number of Type II (**D,E**) and Type III cells (**F,G**) did not differ between mutants and controls. NTPdase2 corrected fluorescence intensity of Type I cells was significantly increased in both YFP^**+**^ and YFP^**-**^ taste buds (**B,C**). Representative stack images and data from 4–5 control and 5–6 mutant mice. **A**: 178 vs 246 YFP^**+**^ taste bud profiles from 6 control mice vs 6 mutant mice, respectively; **B**: 85 vs 64 YFP^**-**^ taste bud profiles and 82 vs 100 YFP^**+**^ taste bud profiles from 4 control mice vs 4 mutant mice, respectively; **D**: 133 vs 122 YFP^**-**^ taste bud profiles and 72 vs 101 YFP^**+**^ taste bud profiles from 4 control mice vs 4 mutant mice, respectively; **F**: 120 vs 93 YFP^**-**^ taste bud profiles and 106 vs 145 YFP^**+**^ taste bud profiles from 5 control mice vs 6 mutant mice, respectively. Mann & Whitney test. Data are represented as scatter plot (individual symbols), and median with interquartile range (blue bars). Nuclei are counterstained with DRAQ5 (blue). Scale bars = 20 μm.

We also assessed cell fate in taste buds of the CVP of mice with β-catenin stabilized in Shh^+^ cells. Unlike the FFP, the number of YFP^+^ Shh-descendent cells per taste bud was increased in the CVP of mutant mice compared to controls (Fig 8A), whereas similar to anterior taste buds, the proportion of taste buds housing YFP^+^ cells was minimally albeit significantly increased in mutant CVP versus controls (S5B Fig). In terms of cell lineage, as shown for FFP, the NTPDase2^+^ Type I cell population in CVP taste buds was increased (Fig 8B and 8C), while the number of Type II and Type III cells in YFP^+^ taste buds was unchanged in mutant CVP compared to controls (Fig 8D–8G). Further, the fate of Shh-descendent cells with cell autonomous activation of β-catenin did not differ between mutants and controls (S1 Table). Unexpectedly, however, in addition to the increase in NTPdase2-IR Type I cells in mutant YFP^+^ taste buds, we also found more Type I cells in YFP-negative taste buds in mutants (Fig 8B and 8C), suggesting that in addition to being promoted locally by signals within taste buds, control of Type I cell differentiation in the CVP is also regulated via signals extrinsic to taste buds.

## Discussion

We showed previously that Wnt/β-catenin signaling is both necessary and sufficient for FFP and early taste bud development during embryogenesis, and moreover, that forced activation of β-catenin induces ectopic and enlarged taste papillae containing enlarged taste buds throughout the lingual epithelium [23]. However, because these embryos died at birth, we were unable to determine if β-catenin also functions in adult taste cell renewal. Here, we show that activation of β-catenin in adult Krt5^+^ lingual epithelial progenitors results in the formation of ectopic taste buds within FFPs and CVPs, and in non-taste lingual epithelia. These data are consistent with the finding that expression of stabilized β-catenin drives formation of ectopic hair follicles in adult epidermis [55], and show that this pathway can determine lingual epithelial, as well as epidermal, fate decisions in the adult. Beta-catenin signaling is also normally active in lingual filiform papillae [25], but at lower levels than in taste buds. Thus our data suggest that high levels of β-catenin signaling, such as those observed in taste papillae, direct adult lingual cells to a taste fate.

Both within and outside taste papillae, we find that activated β-catenin initially triggers hyperproliferation of Krt5^+^ lingual progenitors. This finding is consistent with previous data showing that β-catenin signaling is required for normal levels of proliferation of lingual epithelia [25], and suggest that this pathway drives taste bud renewal. Similarly, β-catenin signaling contributes to progenitor cell proliferation in the epidermis, hair follicles, and intestinal epithelium [25, 56, 57]. Interestingly, following an initial bout of proliferation, taste cells expressing stabilized β-catenin are driven to differentiate, predominantly towards a Type I taste cell fate. These data suggest that, in addition to its pro-proliferative role, β-catenin also drives differentiation of taste epithelium.

Our data parallel findings in skin and intestinal epithelia. In each of these tissues, Wnt signaling plays diverse roles, promoting both proliferation and terminal differentiation. For instance, in the small intestine, Wnt signaling is required for progenitor cell proliferation, and also for acquisition of Paneth cell fate [58, 59]. In hair follicles, Wnt signaling is necessary for proliferation of transit amplifying cells in the secondary hair germ and matrix, and also drives matrix cells to terminally differentiate into hair shaft progenitors [25, 60–62]. The mechanisms underlying these seemingly opposing activities are not fully understood, but may depend in part on the level of activity of the signaling pathway. For instance, in adult hair follicle matrix cells and interfollicular epidermis, relatively low levels of signaling are thought to drive proliferation, while high levels of activity cause cells to terminally differentiate [25]. Although active Wnt/β-catenin signaling is evident in all three differentiated taste cell types [31], we find that stabilization of β-catenin in Krt5^+^ lingual progenitor cells predominantly biases these cells towards a Type I fate, with a lesser induction of Type II taste cell fate, both within and outside existing taste papillae. This differential induction of the different taste cell types may in part reflect differences in their relative proportions in control taste buds, *i*.*e*., I > II > III [19, 20], and that Type III cells are longer lived than Type II cells [16], and thus Type III cells would be predicted to be generated least frequently. However, our data more strongly support a model, as in skin epithelia, in which graded levels of β-catenin signaling determine the precise fate of taste cells, with the highest levels promoting Type I fate, while moderate levels of β-catenin are required for Type II differentiation, and both high and mid levels preclude acquisition of Type III fate. Additionally, β-catenin does not bind DNA directly, but rather forms multiprotein complexes that include Lymphoid Enhancer Factor/T Cell Factor (LEF/TCF) family members and cell-type-specific transcription factors [63]. The combination of cooperating factors expressed in any given cell type is thus likely to determine which of β-catenin’s potential target genes are expressed and to contribute to the precise outcome of pathway activation.

The cell autonomous impact of β-catenin on cell fate appears most robust when activated in the Krt5^+^ progenitor population, while stabilization of β-catenin within Shh^+^ postmitotic precursors does not alter significantly the fate of these cells. However, increased β-catenin signaling within this lineage-labeled subset does influence cell fate indirectly, as mutant taste buds house more Type I cells in both the FFP and CVP than controls. How this shift in cell fates comes about is not known, but may rely on additional signals emitted from these new taste cells with persistent stabilized β-catenin.

One candidate pathway that may mediate these effects is Sonic hedgehog (Shh) signaling. In the tongues of control mice, Shh expression is restricted to intragemmal basal cells (Type IV) within taste buds [36], which are the immediate precursors of each of the 3 differentiated taste cell types [22]; while Shh target genes *Ptch1* and *Gli1*, are expressed by keratinocytes adjacent to taste buds [36]. This expression pattern suggests that SHH from within buds signals to adjacent progenitors to regulate taste cell renewal [21]. Indeed, we have shown recently that Shh promotes taste bud cell differentiation, as ectopic overexpression of SHH induces the formation of ectopic taste buds throughout the non-taste epithelium [41]. However, distinct from the uniform Type I cells induced by β-catenin, SHH-induced ectopic taste buds possess all 3 differentiated taste cell types [41]. Thus, β-catenin appears to act upstream of Shh as stabilized β-catenin induces excess and ectopic clusters of Shh^+^ epithelial cells in the anterior and posterior tongue (Fig 6), suggesting that the Shh^+^ precursor step is obligate for taste cell differentiation in general, yet high β-catenin levels drive Shh^+^ cells to differentiate predominantly as Type I taste cells.

While generally comparable, elements of β-catenin-mediated regulation of taste cell renewal differ between anterior and posterior tongue. Activated β-catenin drives differentiation of Type I and to a lesser extent Type II cells in both taste fields, but sparse Type II cells are evident in the anterior tongue only after prolonged induction. By contrast, both excess Type I and II cells appear rapidly within 4 days in the CVP epithelium. Additionally, stabilization of β-catenin in Shh^+^ precursors induced more Type I cells in both YFP^+^ and YFP^-^ taste buds in the CVP, but in FFP only YFP^+^ buds were affected. This may be due to differences in the embryonic origins of anterior versus posterior taste buds. The posterior tongue, including the CVP, arises from the foregut endoderm [32], while the anterior tongue is ectodermally derived. Although the oral cavity is lined by a continuous epithelium, and taste buds are thought to be homologous regardless of location [64], these commonalities have arisen by different embryonic histories, which are revealed by differences in BMP4 expression in adult taste buds and FGF functional regulation of taste papilla development [65–67]. Likewise, differences in embryonic origin might cause differential region-specific expression of Wnt pathway components, such as Lgr5 [68, 69], which could in turn contribute to the differential response of anterior and posterior taste epithelia to increased β-catenin signaling [70, 71].

While the impact of β-catenin stabilization in Shh^+^ precursors was strictly taste bud autonomous in the anterior tongue, *i*.*e*., only buds with YFP^+^ cells were affected, in the posterior CVP, increased β-catenin under the control of the Shh promoter resulted in more Type I cells in taste buds, regardless of whether the taste bud possessed Shh-descendent YFP^+^ cells or not. This finding implies that stabilized β-catenin in this Shh^+^ cell population(s) may impact CVP taste cell fate more broadly. The anterior FFP are small, simple structures housing single taste buds, and FFP are typically distributed in the lingual epithelium at low density (~100/cm^2^) [72]. The single rodent CVP, by contrast contains hundreds of buds, which are packed in close proximity to one another, interspersed with small numbers of Krt14^+^ cells basolaterally, and Krt13^+^ cells apicolaterally (see Fig 1A, control). Therefore, increased signaling from Shh^+^ cells with activated β-catenin may be detectable by adjacent progenitors and taste buds, and thus impact cell fate decisions indirectly. The nature of this signal remains to be explored.

In conclusion, we show that, in parallel with its roles in skin, intestinal and neural epithelia, forced activation of β-catenin signaling promotes acquisition of taste fate, affects both renewal and differentiation of taste buds in adult mice, and does so primarily at the level of the progenitor population. Because radiotherapy targeting head and neck cancers causes taste dysfunction [73], and taste cell renewal is reduced in mice following head and neck radiation [46], our data suggest canonical Wnt signaling as a potential therapeutic target to restore taste sensitivity in these patients. Our data also suggest that, similar to the effects of Shh pathway antagonists [74], systemic cancer therapeutics that block Wnt signaling may cause taste dysfunction, and would need complementary treatment to help restore normal taste function to avoid malnutrition and psychological distress in these patients.

## Materials and Methods

### Ethics statement

Mice were housed in compliance with the Guide for the Care and Use of Laboratory Animals, Animal Welfare Act and Public Health Service Policy. All procedures were approved by the Institutional Animal Care and Use Committee at the University of Colorado Anschutz Medical Campus.

### Animals and procedures

Male and female transgenic mouse lines were all on a mixed background (FVB, 129Sv, C57Bl6). All experimental tissue was generated from adult mice between 7–11 weeks of age. To stabilize β-catenin in epithelial progenitors of taste and non-taste epithelium, trigenic mice were generated: 1) Krt5rtTA—expression of a transcriptional activator rtTA is controlled by the human Krt5 promoter [75]; 2) tetO-Cre tetracycline-sensitive tetO response element controls production of Cre recombinase [76]; 3) Ctnnb1^(Ex3)fl—^floxed allele of β-catenin with exon 3 flanked by loxP sites [27]. Krt5rtTA;tetOCre;Ctnnb1^(Ex3)fl/+^ mice were fed the tetracycline analog doxycycline ad libitum in their chow (Bio-Serv, Frenchtown, NJ, 1g/kg) continuously until sacrifice at 2–14 days (longer time points were not possible as mice sickened by 16 days and died around 20 days).

To validate our model, we fed Krt5-rtTA;tetO-Cre;R26RLacZ reporter mice [77] doxycycline chow up to 28 days and then examined lingual tissue via Xgal reaction.

To explore whether β-catenin directly regulates the differentiation of precursors into taste cells, we stabilized β-catenin in Shh-expressing cells by generating trigenic mice: 1) ShhCreERT2—expression of an tamoxifen-inducible Cre recombinase under the Shh promoter [78]; 2) Ctnnb1^(Ex3)fl—^[27]; 3) R26R-YFP—expression of YFP in the Rosa locus downstream of a LoxP-flanked stop sequence [79]. Mice were gavaged with tamoxifen (100 mg/kg_bw_; stock solution 10 mg tamoxifen/ml corn oil) once every morning for 8 consecutive days, and were sacrificed 14 days after the last gavage.

### Tissue collection

Tongues were dissected from the lower jaw and quickly frozen, fixed via direct immersion, or following transcardial perfusion with Periodate-lysine-paraformaldehyde (PLP) or 4% paraformaldehyde (PFA) in 1× Phosphate Buffer (PB: 29 mM NaH2PO4, 75 mM Na2HPO4). Fixation method is specified in S2 Table for each antiserum used.

#### Fixed tissue

Mice were anesthetized by i.p. injection of 250 mg/kg_body weight_ Avertin (2,2,2-Tribromoethanol), and ice cold 0.9% sodium chloride was perfused transcardially to clear blood, followed by Periodate-Lysine-Paraformaldehyde (PLP: 75mM L-lysine monohydrochloride, 1.6% paraformaldehyde, 10 mM sodium periodate) or 4% PFA fixative perfusion. Tongues were then incubated for 3 h in fixative at 4°C, and placed in 20% sucrose (Fisher Scientific, Pittsburgh PA, USA) in 1× PB overnight at 4 °C. Samples were embedded in O.C.T Compound (Tissue-Tek 4583, Sakura Finetek, Torrance CA, USA), frozen on dry ice and stored at -80°C.

#### Fresh tissue

Mice were euthanized by CO_2_ inhalation followed by cervical dislocation. The tongues were collected, rinsed in sterile ice-cold 1× Phosphate Buffered Saline (PBS: 29 mM NaH2PO4, 75 mM Na2HPO4, 154 mM NaCl) and embedded in O.C.T Compound, frozen on dry ice and stored at -80°C.

#### Peeled epithelium

Mice were euthanized by CO_2_ inhalation followed by cervical dislocation. The tongues were collected, rinsed in ice-cold Normal Tyrode solution (140 mM NaCl, 5 mM KCl, 10 mM HEPES, 4 mM CaCl2, 10 mM glucose, 1 mM MgCl2, 1 mM sodium pyruvate, pH 7.4), and 200 µl of dispase II (3 mg/ml Normal Tyrode solution) was injected underneath the epithelium. Tongues were incubated in calcium-free Tyrode solution at room temperature for 20 min, the epithelium (tip to intermolar eminence) was peeled, immediately placed in a tube and frozen on dry ice. Samples were stored at -80°C.

### X-Gal reaction

X-gal reactions were performed on 12 μm cryostat PLP-fixed sections collected on Superfrost Plus Slides. Sections were washed with solution 1 (0.02% Nonidet P40, 2 mM Mgcl2 in 1× PBS pH 7.3), and incubated in reaction solution (5 mM Potassium ferrocyanide, 5mM Potassium ferricyanide, 0.5 mg/ml X-gal in solution 1) at 37°C until desired staining was obtained. Slides were washed in 1× PB, and coverslipped using Fluoromount G (SouthernBiotech, Birmingham AL, USA).

### Immunohistochemistry

Immunolabeling was performed on 12 μm cryostat sections collected on Superfrost Plus Slides (Fisher Scientific, Pittsburgh PA, USA) following Gaillard and Barlow (2011) [31]. Sections were rehydrated in 1× PBS prior to staining. Antigen specific protocols are detailed below. Primary and secondary antisera, amplification systems, and dilutions used are listed in S2 Table. Immunoreactivity for each antigen listed was abolished when primary antibodies were omitted. Nuclear counterstain was performed using Sytox Green Nucleic Acid Stain (Invitrogen), or DRAQ5 (Abcam).

#### β-catenin

Sections were incubated in 10 mM sodium citrate pH 6 + 0.05% tween 20 at 95°C for 15 min, and incubated in blocking solution (2% normal goat serum, 1% bovine serum albumin, 0.3% Triton X100 in 1× PBS, pH 7.3) for 45 min at room temperature. Because the antiserum against β-catenin was produced in mouse, we used the M.O.M. kit from Vector Labs (BMK-2202). First, blocking of non-specific binding sites of the avidin/biotin amplification system was performed by using an Avidin/Biotin kit (Vector Labs SP-2001). Sections were incubated in Avidin solution for 15 min, rinsed, incubated in Biotin solution for 15 min and rinsed again. Slides were dipped in M.O.M. Ig blocking reagent for 1h at room temperature, washed in 1× PBS, incubated in M.O.M. diluent (protein concentrate diluted 1/14 in 1× PBS) for 10 min at room temperature, and incubated in primary antiserum raised against β-catenin (diluted in M.O.M. diluent) overnight at 4°C. Sections were washed, incubated in M.O.M. biotinylated anti-mouse IgG reagent (diluted in M.O.M. diluent) for 1 h at room temperature, washed again, and incubated with Streptavidin Alexa 488 diluted in PBS+0.1% Triton X100 1 h at room temperature. Nuclei were counterstained with DRAQ5 (Abcam, Cambridge, UK) diluted 1/8000 in 1× PB for 30 min at room temperature. Slides were coverslipped using Fluoromount G.

#### Cell types, keratins, Skn-1a, Mash1, Claudin4, YFP

Fresh tissue or PLP-fixed sections were thawed and post-fixed in 4% PFA for 10 min at room temperature, washed with 1× PBS, incubated for 1.5 h at room temperature in blocking solution (5% normal goat serum, 1% bovine serum albumin, 0.3% Triton X100 in 1× PBS, pH 7.3), and incubated with primary antisera diluted in blocking solution, overnight at 4°C (4 days for MASH1[80]). Sections were washed prior to incubation with secondary antisera diluted in blocking solution for 1 h at room temperature (2 h for MASH1), and washed in 1× PBS. Triple stained fresh tissue sections for Krt8/Krt13/Krt14 were fixed in 4% paraformaldehyde for 10 min at room temperature, and washed in 0.1M PBS. Fixed and fresh sections were counterstained with DRAQ5 as described above, and coverslipped using Fluoromount G.

#### Ki67

Sections were incubated in 10 mM sodium citrate pH 6 + 0.05% tween 20 at 95°C for 15 min. Once slides reached room temperature, sections were incubated in blocking solution for 1 h at room temperature, then incubated with Ki67 antiserum diluted in blocking solution overnight at 4°C. Sections were rinsed, and blocking of non-specific binding sites of the avidin/biotin amplification system was performed by using an Avidin/Biotin kit (Vector Labs SP-2001), as above. Sections were incubated with the anti-rabbit biotin-conjugated antibody diluted in 1× PBS, 0.1% tween 20, 2.5% normal goat serum for 1 h at room temperature. Samples were then incubated in streptavidin-Alexa 546 diluted in 1% bovine serum albumin, 0.3% Triton X100 in 1× PBS, pH 7.3 for 1 h at room temperature, rinsed, counterstained with SYTOX green (Invitrogen) diluted 1/30000 in 1× PB pH 7.2 for 1 min at room temperature, rinsed again, and slides were mounted with Fluoromount G.

### TUNEL assay

To assess cell death, the In Situ Cell Death Detection Kit TMR red (Roche Applied Science, Cat #12156792910) was used. Sections were washed in 0.1M PBS prior to antigen retrieval in 0.1 M Sodium Citrate pH = 6 for 15 min at 90°C. Sections were washed and incubated in a permeabilization solution (0.1% Triton X100 in 0.1% Sodium citrate) for 2 min on ice. Slides were washed and incubated in blocking buffer (50 mM Tris-HCl pH = 7.5, 3% BSA, 20% NGS) 30 min at room temperature. TUNEL reaction was performed according to the manufacturer’s instructions, by mixing 1 volume of Enzyme Solution with 9 volumes of Label Solution, and incubating the sections 60 min at 37°C in humidified atmosphere. Sections were washed, counterstained with Sytox Green, and slides mounted with Fluoromount G. One negative control was included by incubating a slide with the Label Solution only, and one positive control was added by incubating a slide with DNase I (10 U/ml in 50 mM Tris-HCl pH 7.5, 1 mg/ml BSA) 20 min at room temperature prior to executing the TUNEL reaction.

### *In situ* hybridization

Detection of mRNA encoding for *Shh* was performed as previously described [31]. Antisense RNA probes were synthesized from a linearized plasmid containing a *Shh* cDNA insert [81], *Ptch1* cDNA insert (1318–2362: Genbank MMU46155), or *Mash1* cDNA insert (10012: Genbank U68534–783: Genbank M65603, 1276 bp), using FITC-conjugated UTP or digoxigenin-conjugated UTP. Sections were incubated in 4% PFA for 10 min at room temperature, rinsed in 0.1× PBS (14 mM NaCl, 0.3 mM KCl, 0.3 mM Na2HPO4, 0.2 mM KH2PO4), incubated in triethanolamine solution (1.3% triethanolamine, 0.175% HCl 10 N, 0.25% acetic anhydride), rinsed in 0.1× PBS, incubated in hybridization solution (50% formamide, 5× SCC (750 mM NaCl, 75 mM sodium citrate dihydrate), 5× Denhardt’s solution (0.1% Ficoll, 0.1% polyvinylpyrrolidone, and 0.1% bovine serum albumin), 500 μg/ml salmon sperm DNA and 250 μg/ml tRNA) for 2 h at room temperature, then with the RNA probes in hybridization solution overnight at 65°C in a moist chamber. Sections were incubated 90 min at 65°C in 0.2× SSC (30 mM NaCl, 3 mM sodium citrate dihydrate), then in Buffer 2T for 1 h at room temperature, and incubated with peroxidase-coupled anti-digoxigenin antibody diluted 1/600 or alkaline phosphatase-coupled anti-FITC antibody diluted 1/5000 in Buffer 2T overnight in a moist chamber at 4°C. To detect *Shh* mRNA in the CVP, sections were treated with Streptavidin-Alexa 488 diluted 1/400 (Invitrogen, Carlsbad, CA, USA) for 30 min following a 30 min tyramide-biotin treatment (TSA Biotin Tyramide Reagent, PerkinElmer, Waltham, MA, USA). In the anterior tongue, *Shh*, *Patched1* and *MASH1* mRNA transcripts were detected by incubating sections with NBT/BCIP solution (Roche Applied Science, 11681451001) in Buffer 3 (0.1 M Tris-HCl pH 9.5, 0.1 M NaCl, 50 mM MgCl_2_) at room temperature until desired staining is obtained. Reaction was blocked in Buffer 4 (10 mM Tris-HCl pH 8, 1 mM EDTA) for at least 10 min, and slides were coverslipped with Fluoromount G.

### Real-time RT-PCR

Total RNA was extracted from peeled anterior tongue epithelium using the RNeasy Plus Mini kit (Qiagen). Bioanalyzer 2100 (Agilent technologies) was used to assess RNA integrity. cDNA was prepared by Reverse Transcription of 1 μg total RNA using the Omniscript Reverse Transcription kit (Qiagen). *Mash1* mRNA levels were normalised to β-actin mRNA levels. cDNA equivalent of 20 ng total RNA, 250 nM of the forward and reverse primers were mixed with the Power SYBR Green PCR Master Mix (Applied Biosystems). Primers sequences were as follows: *Mash1* (NM_008553)[82]: forward 5’-GCAACCGGGTCAAGTTGGT-3’, reverse 5’-GTCGTTGGAGTAGTTGGGGG-3’; β-actin (NM_007393)[83]: forward 5’-ACCAACTGGGACGATATGGAGAAGA-3’, reverse 5’-TACGACCAGAGGCATACAGGGACAA-3’. Real-time PCR consisted of forty 95°C/15 s-60°C/60 s cycles. The comparative ΔΔCt method was used for relative quantification of gene expression [84].

### Scanning electron microscopy (SEM)

Scanning electron microscope experiments were performed at CDB/CVI Microscopy Core (Perelman School of Medicine, University of Pennsylvania). Tongue samples were washed three times with 1× PBS, fixed overnight in 4% PFA and dehydrated in a graded series of ethanol concentrations through 100% over a period of 1.5 hour. Dehydration in 100% ethanol was done three times. Dehydrated samples were then incubated for 20 min in 50% HMDS in ethanol followed by three changes of 100% HMDS (Sigma-Aldrich Co.) and followed by overnight air-drying as described previously [85]. Then samples were mounted on stubs and sputter coated with gold palladium. Specimens were observed and photographed using a Quanta 250 scanning electron microscope (FEI, Hillsboro, OR, USA) at 10 kV accelerating voltage.

### Image acquisition and analysis

Confocal fluorescence images were acquired using a Leica TCS SP5 II laser-scanning confocal microscope and LASAF software. Nomarski images were acquired using a Zeiss Axioplan 2 microscope, camera and software. All sections of the CVP, except the first and last sections which were excluded as they generally contain incomplete trenches, were analyzed. For the anterior tongue, 12 μm serial sections were cut into 6 sets such that sections on each slide were separated by 72 μm. FFP were analyzed in the 3^rd^ through the 12^th^ section, while the 1^st^ and 2^nd^ sections were omitted due to the curved nature of the tongue surface and difficulty in interpreting non-transverse sections through FFP. Thus we analyzed FFP in a region representing 720 μm of the anterior tongue starting ~145 μm from the tongue tip. Proliferative index (P.I.) in the CVP was calculated by dividing the number of Ki67^+^ basal cells by the number of Sytox Green^+^ basal cells, *i*.*e*., cells residing along the basement membrane, within the portion of the CVP trenches housing taste buds [46]. In the anterior tongue, the number of Ki67^+^ cells was tallied along 400 μm of non-taste epithelium on the dorsal part of the tongue.

ImageJ (NIH) was used to measure the corrected integrated density of Krt8-, Krt14-, PLCβ2- and NTPdase2-immunofluorescence signal. NTPdase2 is also expressed by Schwann cells of the CVP innervation (green) [49], but this component of NTPdase2^+^ signal was excluded from measurements of epithelial signal, as nerve fibers within taste buds are not myelinated [86]. The area, mean gray value and integrated density were measured in the area of interest, and in 4 small areas selected as the background signal. The corrected integrated density was calculated as follows:

Corrected integrated density = Integrated density − (Area selected × Mean value of background) [87].

Immunolabeled cells were tallied by analyzing both 0.75 μm optical sections and compressed z-stacks (14 optical sections). Immunolabeled cells and *in situ* labeled cells were counted when a nuclear profile was identifiable (nuclear staining, counterstain, or no staining in an elliptical shape within a cytoplasmically stained cell of interest).

### Statistical analysis

Statistical analyses were performed using SigmaStat (Systat Software). Normal distribution and equal variances between groups were assessed with a p value set at 5%, to determine whether to run a Mann-Whitney test or a Student’s t-test. Statistical differences were established with a minimum confidence interval of 95%. Non-parametric data are represented as medians, 1^st^ and 3^rd^ quartiles, and error bars represent minimum and maximum values, while parametric data are represented as means ± SEM. Sample sizes for data are presented in the figure legends.

## Supporting Information

S1 Fig Validation of the mouse model genetics. To validate the doxycycline-mediated Krt5-driven induction system, mice carrying the Krt5rtTA;tetOCre alleles were crossed with Rosa26-LacZ reporter mice to map the fate and kinetic of the Krt5^+^ cells. X-Gal reaction was performed on CVP and FFP sections from mice carrying the 3 alleles (Krt5rtTA;tetOCre;Rosa-LacZ). In the absence of doxycycline, β-galactosidase was not expressed in either basal cells or their descendants (**A**_**1**_,**B**_**1**_), demonstrating that expression of the Cre recombinase does not leak. When trigenic mice were fed doxycycline for 4 days, most perigemmal basal cells expressed β-galactosidase, while only a few Krt5-descendant cells had entered taste buds (**A**_**2**_,**B**_**2**_, white arrowheads). More Krt5-descendant cells were evident in taste buds after 7 days and 4 weeks of doxycycline (**A**_**3–4**_,**B**_**3–4**_). To ascertain that the β-catenin GOF was effective in the CVP and FFP of trigenic mice fed doxycycline, sections were immunostained with antiserum against β-catenin. β-catenin IR in the cytoplasm and nuclei of taste bud cells was dramatically enhanced in the CVP and FFP in the GOF compared to controls (**C**_**,**_ **D**, white arrowheads. 0.75μm optical confocal sections). Nuclei were counterstained with DRAQ5 in blue. White dash line shows taste buds/taste area. Three mice were used in each experimental group. Scale bars = 20 μm.
(TIF)

S2 Fig *Ptch1* expression in the CVP epithelium is lost in β-catenin GOF mice. *In situ* hybridization for *Ptch1*, which is normally restricted to the progenitor cell compartment (Control), revealed that *Ptch1* expression is virtually absent in the CVP of the β-catenin GOF mice (GOF 4 days). Black dotted line indicates basement membrane, dash line in GOF delimits the expanded taste epithelium. Three mice were used in each experimental group. Scale bars = 20 μm.
(TIF)

S3 Fig Quantification and characterization of the NTPdase2-IR cell population in the CVP and FFP. We used corrected NTPDase2 immunofluorescence intensity as a proxy for the number of NTPdase2^+^ cells. **A.** In the CVP, the epithelial area occupied by NTPdase2^+^ immunofluorescent cells increased nearly 2-fold in mutants compared to controls. The thickness of the NTPDase2^+^ CVP epithelium also increased significantly in GOF mice. NTPdase2^+^ surface area was measured in sections of 7 and 6 CVP trenches from control and GOF mice, respectively. NTPdase2^+^ epithelium thickness was measured in 65 taste buds from 7 CVP trenches in control mice, and 6 CVP trenches in mutant mice. To validate corrected fluorescence intensity as a reliable measure of taste cell number, we applied this method to PLCβ2^+^ Type II cells. We found a significant correlation between the number and the fluorescence intensity of PLCβ2^+^ Type II cells (**B**, left panel, Pearson correlation coefficient r^2^ = 0.683, p = 0.0013, n = 19), and that PLCβ2 immunoreactivity was significantly higher in mutant CVP trenches than in controls (**B**, right panel, p = 0.00002, Student’s t-test, n = 9 control trenches and 10 mutant trenches). In the anterior tongue, β-catenin GOF induced multiple ectopic Krt8^+^ cell clusters within FFP after 7 days on doxycycline and all of these taste bud-like structures were exclusively NTPdase2^+^. Various conformations were observed in the FFP: one large taste bud, duplicates, triplicates or more, were observed in both the apex and base of FFP (**C**). Three mice were used in each experimental group. Student’s t-test. Nuclei were counterstained with DRAQ5 in magenta. Scale bars = 20 μm.
(TIF)

S4 Fig Ectopic taste buds cells induced by stabilized β-catenin for 7 days are exclusively Type I cells. Induction of β-catenin for 7 days triggered the production of ectopic Krt8^+^ taste buds (red) found interspersed among filiform papillae of the non-taste epithelium. These ectopic taste buds never contained SNAP25^+^ type III (left top, green) or PLCβ2^+^ type II (left middle, green) cells, but were readily detected as NTPdase2^+^ (left bottom, green). Nuclei were counterstained with DRAQ5 in blue. Dotted line delimits the basement membrane. Representative stack images and data from 3 control and 3 mutant mice. Scale bars = 20 μm.
(TIF)

S5 Fig Beta-catenin stabilization in Shh^+^ precursors increases the number of taste buds with YFP^+^ cells in the FFP and CVP. ShhCreERT2;Ctnnb1^(Ex3)fl/+^;R26R-YFP mice and their control counterparts (ShhCreERT2;R26R-YFP) were given tamoxifen by gavage daily for 8 days, and tongues harvested 14 days after the last gavage. proportion of taste buds with YFP^+^ cells increased in mutants in both the FFP (**A**), and the CVP (**B**). **A**: 73 vs 79 sections from 6 control mice vs 6 mutant mice, respectively; **B**: 70 vs 68 trench profiles from 6 control mice vs 6 mutant mice, respectively. Mann & Whitney test. Data are represented as scatter plot (individual symbols), and median with interquartile range (blue bars). Scale bars = 20 μm.
(TIF)

S1 Table The number of lineage-labeled Type II and III cells in taste buds in the FFP and CVP does not differ between control (ShhCreERT2;R26R-YFP) and mutant (ShhCreERT2;Ctnnb1^(Ex3)fl/+^;R26R-YFP) mice. (DOC)

S2 Table Primary and secondary antibodies used for immunohistochemistry. (DOC)
