# Supplementary figures and images for: β-Catenin Signaling Biases Multipotent Lingual Epithelial Progenitors to Differentiate and Acquire Specific Taste Cell Fates
Source: PLoS Genet. 2015 May 28;11(5):e1005208. doi: 10.1371/journal.pgen.1005208 (PMC4447363; doi:10.1371/journal.pgen.1005208)

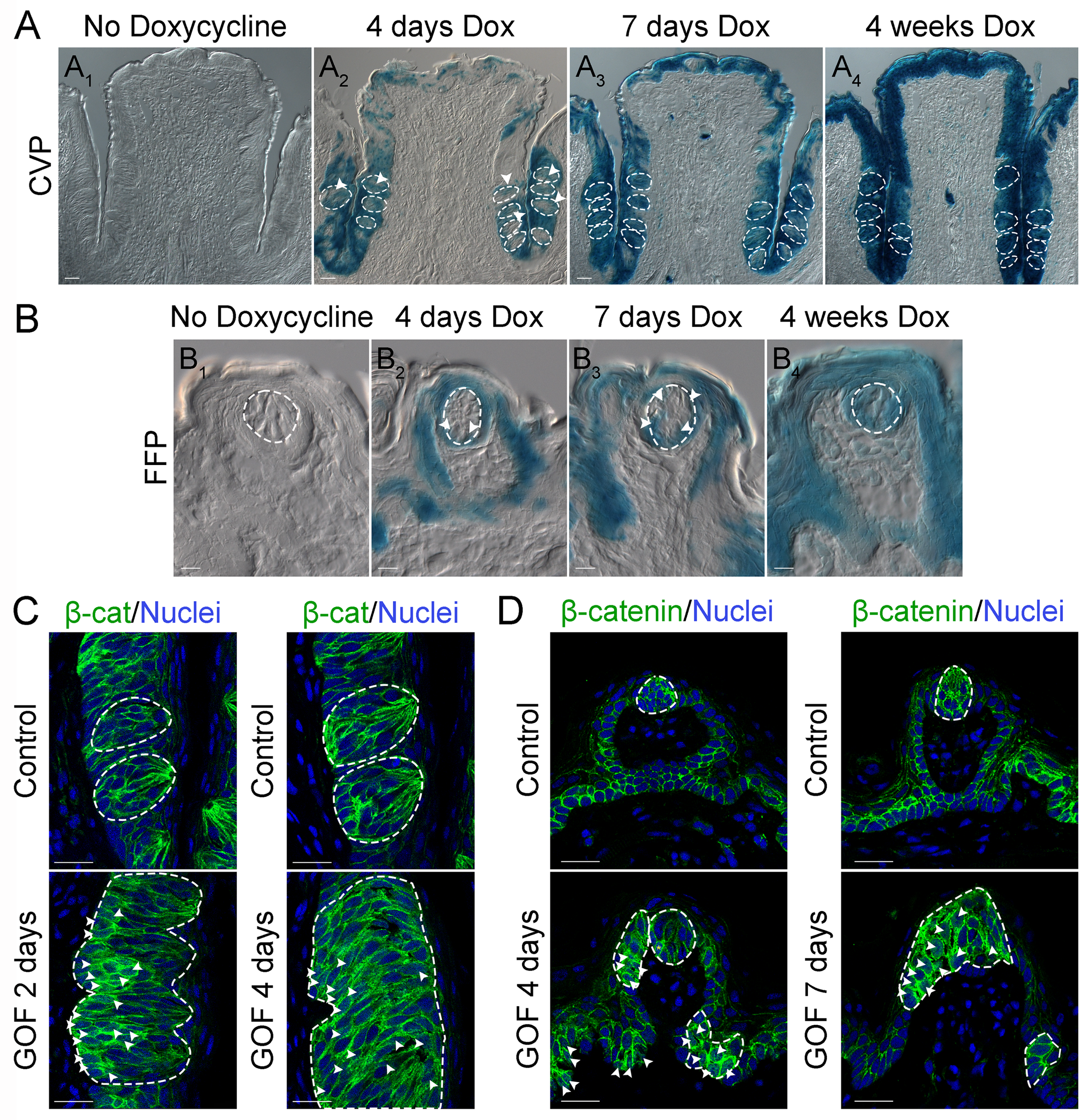

Supplement: S1 Fig — To validate the doxycycline-mediated Krt5-driven induction system, mice carrying the Krt5rtTA;tetOCre alleles were crossed with Rosa26-LacZ reporter mice to map the fate and kinetic of the Krt5+ cells. X-Gal reaction was performed on CVP and FFP sections from mice carrying the 3 alleles (Krt5rtTA;tetOCre;Rosa-LacZ). In the absence of doxycycline, β-galactosidase was not expressed in either basal cells or their descendants (A 1,B 1), demonstrating that expression of the Cre recombinase does not leak. When trigenic mice were fed doxycycline for 4 days, most perigemmal basal cells expressed β-galactosidase, while only a few Krt5-descendant cells had entered taste buds (A 2,B 2, white arrowheads). More Krt5-descendant cells were evident in taste buds after 7 days and 4 weeks of doxycycline (A 3–4,B 3–4). To ascertain that the β-catenin GOF was effective in the CVP and FFP of trigenic mice fed doxycycline, sections were immunostained with antiserum against β-catenin. β-catenin IR in the cytoplasm and nuclei of taste bud cells was dramatically enhanced in the CVP and FFP in the GOF compared to controls (C , D, white arrowheads. 0.75μm optical confocal sections). Nuclei were counterstained with DRAQ5 in blue. White dash line shows taste buds/taste area. Three mice were used in each experimental group. Scale bars = 20 μm. (TIF) [file pgen.1005208.s001.tif]

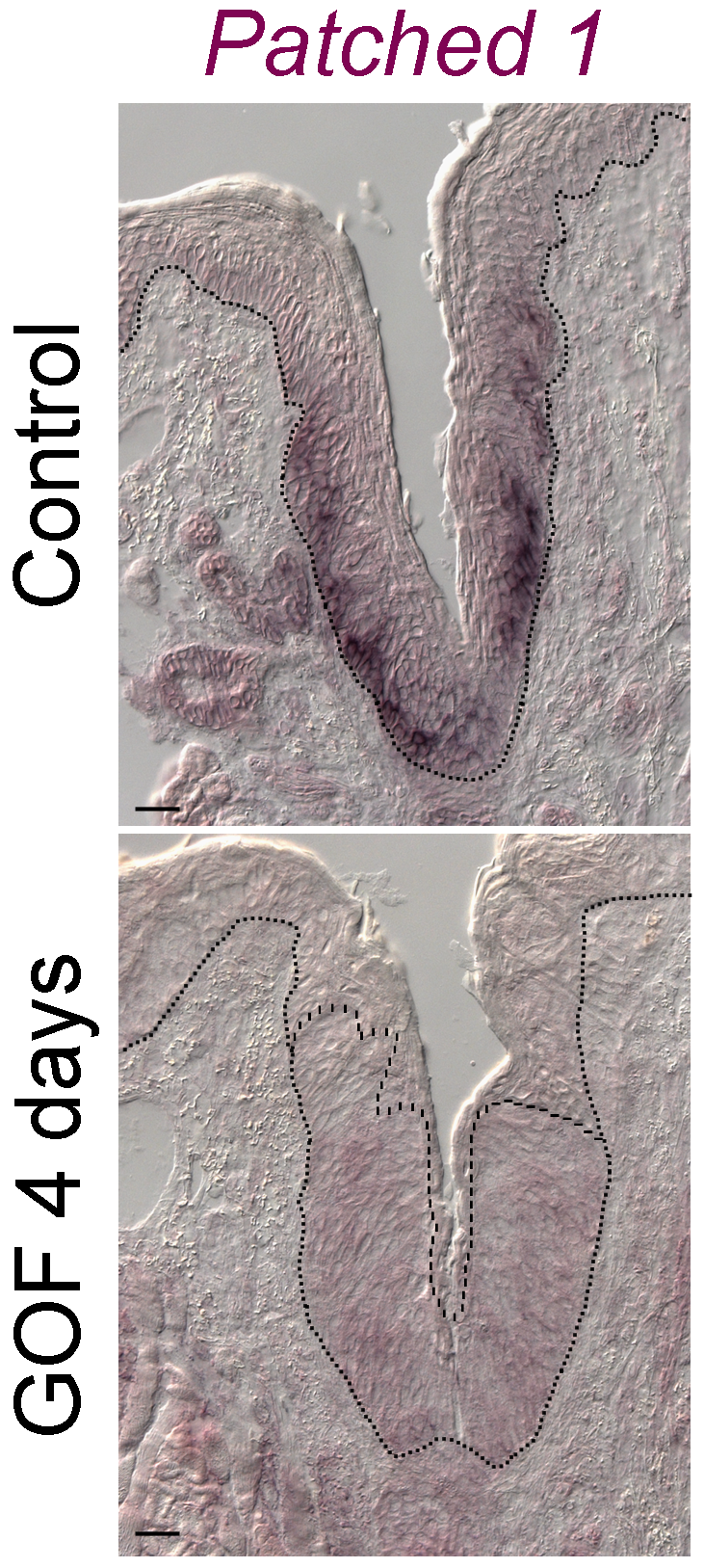

Supplement: S2 Fig — In situ hybridization for Ptch1, which is normally restricted to the progenitor cell compartment (Control), revealed that Ptch1 expression is virtually absent in the CVP of the β-catenin GOF mice (GOF 4 days). Black dotted line indicates basement membrane, dash line in GOF delimits the expanded taste epithelium. Three mice were used in each experimental group. Scale bars = 20 μm. (TIF) [file pgen.1005208.s002.tif]

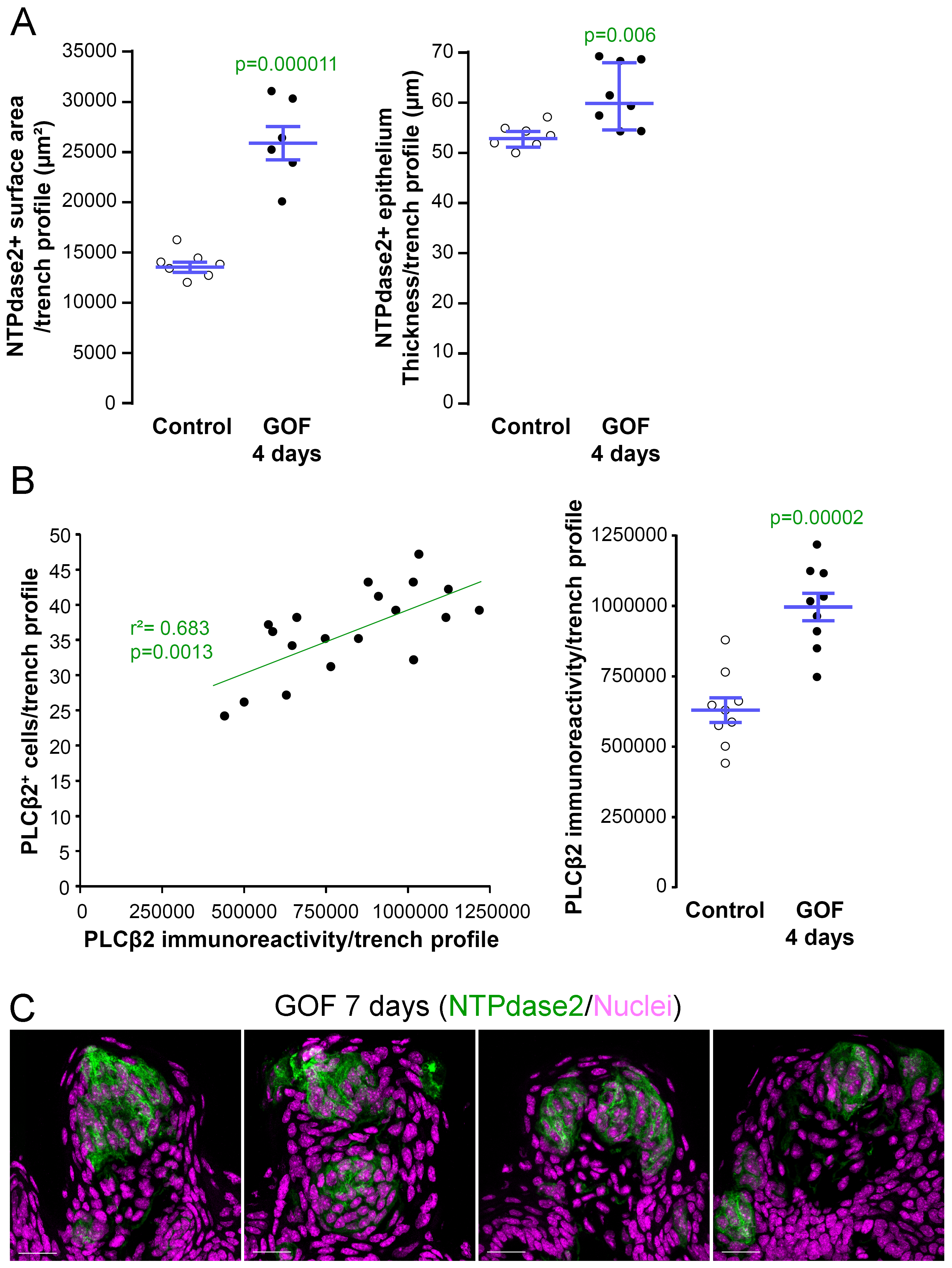

Supplement: S3 Fig — We used corrected NTPDase2 immunofluorescence intensity as a proxy for the number of NTPdase2+ cells. A. In the CVP, the epithelial area occupied by NTPdase2+ immunofluorescent cells increased nearly 2-fold in mutants compared to controls. The thickness of the NTPDase2+ CVP epithelium also increased significantly in GOF mice. NTPdase2+ surface area was measured in sections of 7 and 6 CVP trenches from control and GOF mice, respectively. NTPdase2+ epithelium thickness was measured in 65 taste buds from 7 CVP trenches in control mice, and 6 CVP trenches in mutant mice. To validate corrected fluorescence intensity as a reliable measure of taste cell number, we applied this method to PLCβ2+ Type II cells. We found a significant correlation between the number and the fluorescence intensity of PLCβ2+ Type II cells (B, left panel, Pearson correlation coefficient r2 = 0.683, p = 0.0013, n = 19), and that PLCβ2 immunoreactivity was significantly higher in mutant CVP trenches than in controls (B, right panel, p = 0.00002, Student’s t-test, n = 9 control trenches and 10 mutant trenches). In the anterior tongue, β-catenin GOF induced multiple ectopic Krt8+ cell clusters within FFP after 7 days on doxycycline and all of these taste bud-like structures were exclusively NTPdase2+. Various conformations were observed in the FFP: one large taste bud, duplicates, triplicates or more, were observed in both the apex and base of FFP (C). Three mice were used in each experimental group. Student’s t-test. Nuclei were counterstained with DRAQ5 in magenta. Scale bars = 20 μm. (TIF) [file pgen.1005208.s003.tif]

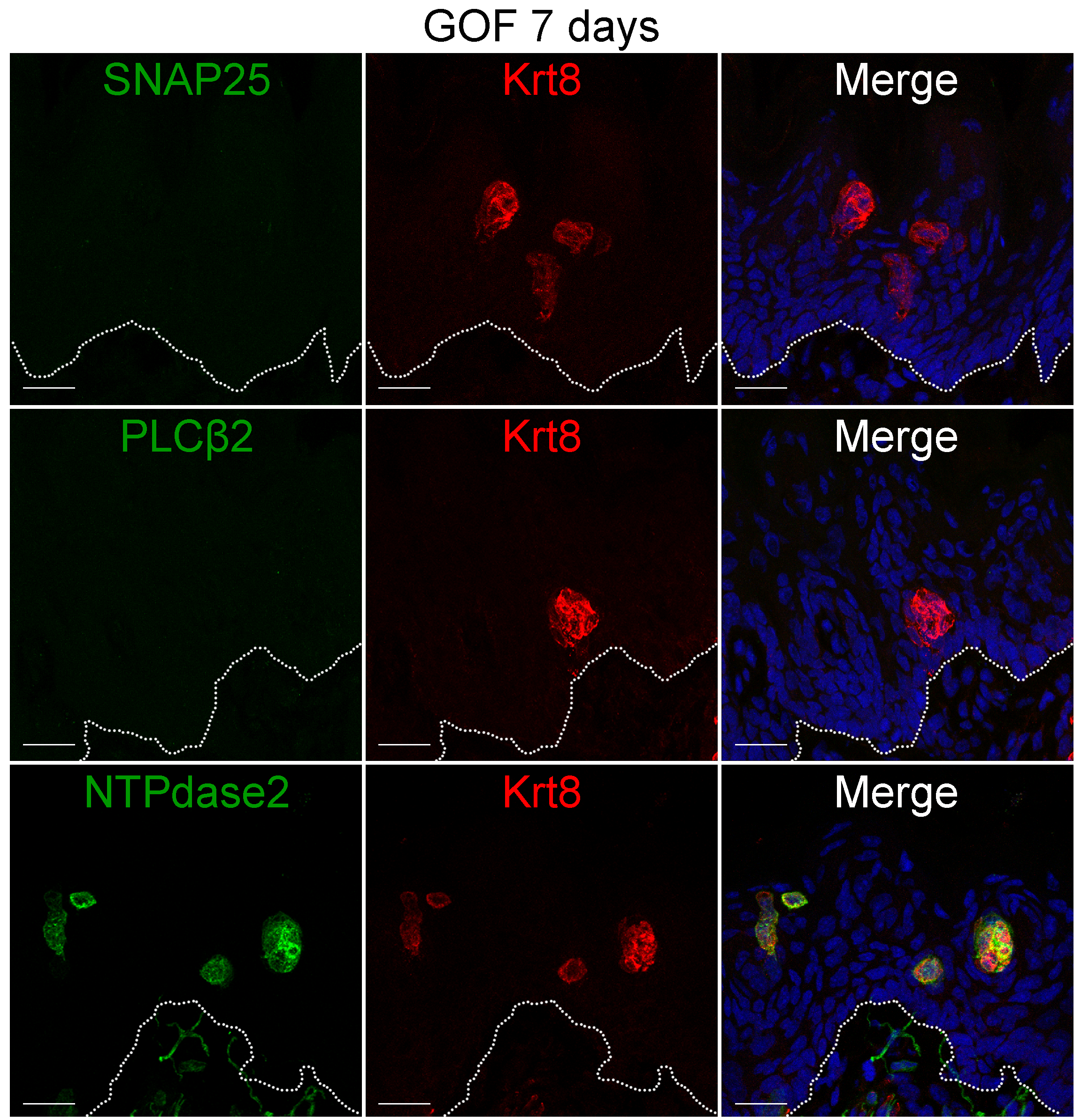

Supplement: S4 Fig — Induction of β-catenin for 7 days triggered the production of ectopic Krt8+ taste buds (red) found interspersed among filiform papillae of the non-taste epithelium. These ectopic taste buds never contained SNAP25+ type III (left top, green) or PLCβ2+ type II (left middle, green) cells, but were readily detected as NTPdase2+ (left bottom, green). Nuclei were counterstained with DRAQ5 in blue. Dotted line delimits the basement membrane. Representative stack images and data from 3 control and 3 mutant mice. Scale bars = 20 μm. (TIF) [file pgen.1005208.s004.tif]

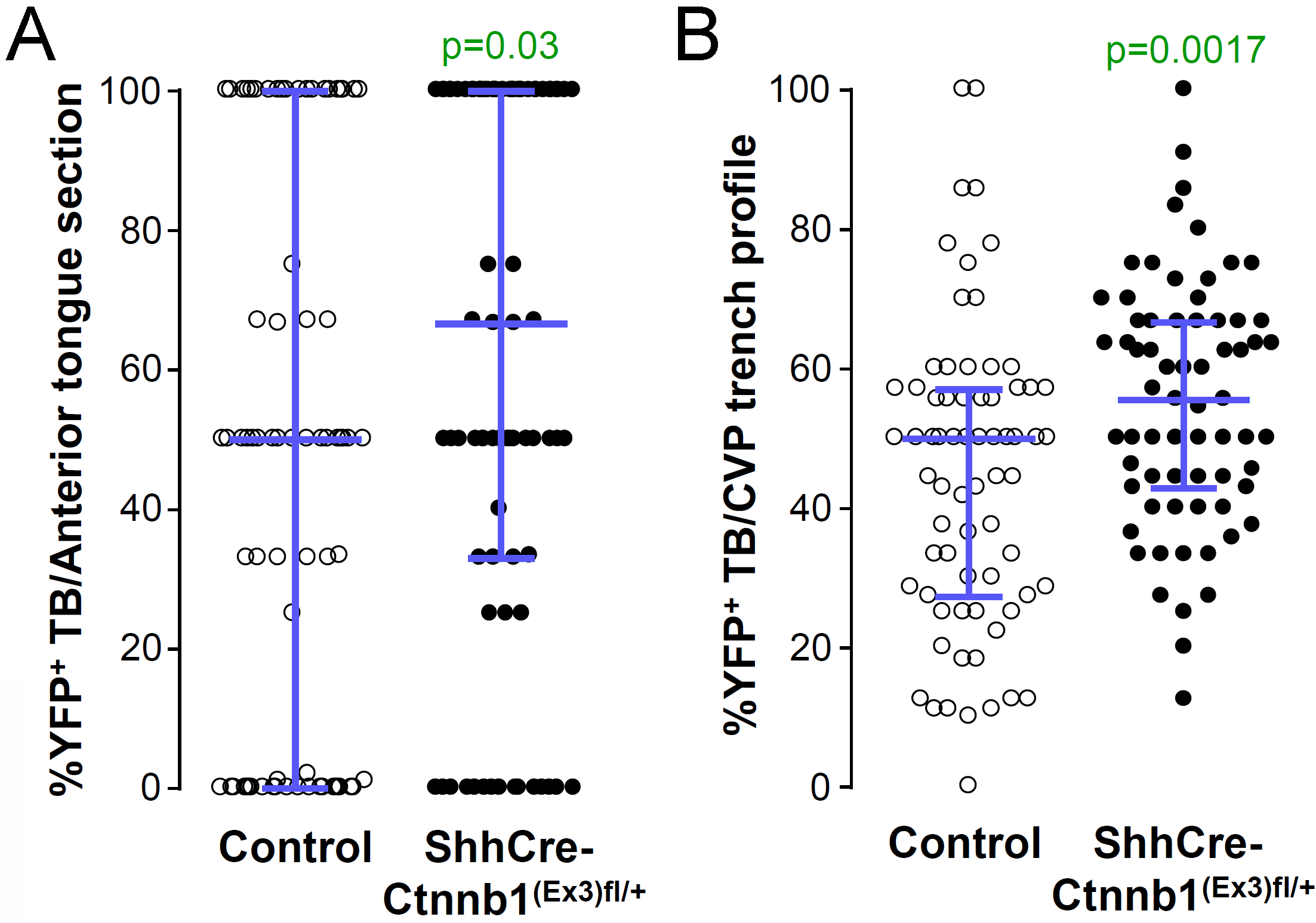

Supplement: S5 Fig — ShhCreERT2;Ctnnb1(Ex3)fl/+;R26R-YFP mice and their control counterparts (ShhCreERT2;R26R-YFP) were given tamoxifen by gavage daily for 8 days, and tongues harvested 14 days after the last gavage. proportion of taste buds with YFP+ cells increased in mutants in both the FFP (A), and the CVP (B). A: 73 vs 79 sections from 6 control mice vs 6 mutant mice, respectively; B: 70 vs 68 trench profiles from 6 control mice vs 6 mutant mice, respectively. Mann & Whitney test. Data are represented as scatter plot (individual symbols), and median with interquartile range (blue bars). Scale bars = 20 μm. (TIF) [file pgen.1005208.s005.tif]
