# Supplementary material for: β-Catenin Signaling Biases Multipotent Lingual Epithelial Progenitors to Differentiate and Acquire Specific Taste Cell Fates
Source: PLoS Genet. 2015 May 28;11(5):e1005208. doi: 10.1371/journal.pgen.1005208 (PMC4447363; doi:10.1371/journal.pgen.1005208)
Supplement: S1 Table — (DOC) [file pgen.1005208.s006.doc]

**S1 Table: The number of lineage-labeled Type II and III cells in taste buds in the FFP and CVP does not differ between control (ShhCreERT2;R26R-YFP) and mutant (ShhCreERT2;Ctnnb1^(Ex3)fl/+^**;R26R-YFP) mice.

|  | **AT** | | | | **CVP** | | | |
| --- | --- | --- | --- | --- | --- | --- | --- | --- |
|  | **PLCβ2/YFP** | | **SNAP25/YFP** | | **PLCβ2/YFP** | | **SNAP25/YFP** | |
|  | **Control** | **ShhCre-Ctnnb1^(Ex3)Fl/+^** | **Control** | **ShhCre-Ctnnb1^(Ex3)Fl/+^** | **Control** | **ShhCre-Ctnnb1^(Ex3)Fl/+^** | **Control** | **ShhCre-Ctnnb1^(Ex3)Fl/+^** |
| **Mean** | 0.36 | 0.17 | 0.24 | 0.12 | 0.19 | 0.20 | 0.38 | 0.41 |
| **SEM** | 0.11 | 0.07 | 0.09 | 0.05 | 0.05 | 0.04 | 0.05 | 0.05 |
| **n** | 4 | 4 | 5 | 6 | 4 | 4 | 5 | 6 |
| **p*** | 0.161 | | 0.203 | | 0.839 | | 0.991 | |

*Mann-Whitney
