# Supplementary material for: β-Catenin Signaling Biases Multipotent Lingual Epithelial Progenitors to Differentiate and Acquire Specific Taste Cell Fates
Source: PLoS Genet. 2015 May 28;11(5):e1005208. doi: 10.1371/journal.pgen.1005208 (PMC4447363; doi:10.1371/journal.pgen.1005208)
Supplement: S2 Table — (DOC) [file pgen.1005208.s007.doc]

|  | **Primary antibody** | **Source**  ***Reference #*** | **Dilution** | **Secondary Antibody** | **Source**  ***Ref #*** | **Dilution** | **Fixation** |
| --- | --- | --- | --- | --- | --- | --- | --- |
| Activated Wnt signaling | **Mouse anti-β-catenin** | Sigma-Aldrich  *C7207* | 1/500 | M.O.M. biotinylated anti-mouse IgG | Vector Labs  *BMK-2202* | 1/250 | PLP |
|  |  |  |  | Streptavidin Alexa Fluor® 488 conjugate | Invitrogen  *S-11223* | 1/600 |  |
| Taste cell precursors | **Guinea pig anti-MASH1** | Tx518  Jane Johnson, UT Southwestern Medical Center  [80] | 1/10000 | DyLight 488 donkey anti-guinea pig | Jackson Immuno Research  *706486148* | 1/800 | Fresh |
|  | **Rabbit anti-Skn1a (Pou2f3)** | Santa Cruz  *sc-330* | 1/500 | Alexa Fluor® 488 goat anti-rabbit IgG | Invitrogen *A11008* | 1/1000 | PLP |
| Cell polarity | **Rabbit anti-Claudin 4** | Invitrogen  *36-4800* | 1/250 |  |  |  | PLP |
| Taste Cell Markers | **Rabbit**  **anti-NTPdase2** | J. Sévigny, Université Laval, Canada [49]  *mN2-36_L_* | 1/3000 |  |  |  | PLP |
|  | **Rabbit**  **anti-PLCβ2** | Santa Cruz  *sc-206* | 1/1000 |  |  |  |  |
|  | **Rabbit**  **anti-SNAP25** | Sigma-Aldrich  *S9684* | 1/6000 |  |  |  |  |
| Cytokeratins | **Rat anti-Krt8** | Developmental Studies Hybridoma Bank  *TROMA-I* | 1/500 | Alexa Fluor® 546 goat anti-rat IgG | Invitrogen *A11081* | 1/1000 | PLP or Fresh |
|  | **Guinea Pig anti-Krt13** | Acris Antibodies  *BP5076* | 1/1000 | Alexa Fluor® 488 goat anti-guinea pig IgG | Invitrogen *A11073* | 1/1000 | Fresh |
|  | **Rabbit anti-Krt14** | Covance  *PRB-155P* | CVP: 1/2500  FFP: 1/3500 | Alexa Fluor® 647 goat anti-rabbit IgG | Invitrogen *A21245* | 1/1000 | Fresh |
| Proliferation Marker | **Rabbit anti-Ki67** | Thermo Scientific  *RM-9106-S* | 1/200 | Anti-rabbit IgG biotinylated | Vector Labs  *PK-6101* | 1/500 | PLP |
|  |  |  |  | Streptavidin Alexa Fluor® 546 conjugate | Invitrogen  *S11225* | 1/1000 |  |
|  | **Mouse anti-BrdU** | Roche  *11 170 376 001* | 1/50 | Alexa Fluor® 488 goat anti-mouse IgG | Invitrogen *A11001* | 1/1000 | Fresh |
| Fate mapping R26R-YFP | **Chicken anti-GFP** | Millipore  *AB16901* | 1/1000 | Alexa Fluor® 488 goat anti-chicken IgG | Invitrogen *A11039* | 1/1000 | PLP |

**S2 Table: Primary and secondary antibodies used for immunohistochemistry**

*PLP: Periodate-Lysine-Paraformaldehyde (see Methods)
